# Conservation Status, Plastome Diversity, and Evolutionary Diversification of Three Arabian *Desmidorchis* Endemics (Apocynaceae)

**DOI:** 10.3390/biology15100798

**Published:** 2026-05-17

**Authors:** Samah A. Alharbi, Othman S. S. Al-Hawshabi

**Affiliations:** 1 Department of Biology, Faculty of Science, Umm Al-Qura University, Makkah 24381, Saudi Arabia; 2 Biology Department, Faculty of Science, University of Aden, Khormaksar, Aden P.O. Box 6235, Yemen; outhman.sad.scie@aden-univ.net

**Keywords:** Arabian Peninsula, codon usage bias, comparative plastome genomics, cpSSR, *Desmidorchis*, IUCN Red List, molecular dating, purifying selection, Stapeliinae

## Abstract

The genus *Desmidorchis* Ehrenb. (Apocynaceae) is a characteristic component of the succulent flora of the Arabian Peninsula, where high levels of endemism and increasing environmental pressures highlight the need for integrated genomic and conservation research. This study assessed the conservation status of three ethnomedicinally important endemics—*D. adenensis*, *D. arabica*, and *D. awdeliana*—and characterizes their complete plastomes to resolve their evolutionary and temporal history. Conservation assessments were conducted following IUCN Red List criteria, and complete plastomes were sequenced and compared within a dataset of 15 subtribe Stapeliinae taxa. Comparative analyses examined the genome structure, divergence hotspots, repetitive sequences, codon usage bias, and selection pressure, while divergence times were estimated using fossil-calibrated molecular clock analyses. All three species were classified as Near Threatened (NT), primarily due to anthropogenic and environmental pressures. Plastome analyses revealed a highly conserved genome structure; however, hypervariable regions, particularly *ycf1* and *clpP1*, exhibited elevated sequence divergence and phylogenetic informativeness. Simple sequence repeats (SSRs) were also identified as potentially informative features at the genus level. Codon usage and Ka/Ks analyses further indicated that most plastid protein-coding genes are under strong purifying selection, whereas only a few loci, particularly *clpP1*, showed comparatively elevated evolutionary rates. Phylogenomic analyses supported the monophyly of *Desmidorchis*, with molecular dating indicating recent Pleistocene diversification (~0.34–1.51 Ma), potentially associated with Quaternary climatic oscillations. Overall, this study provides an important genomic foundation for future taxonomic, evolutionary, and conservation studies of rare Arabian taxa.

## Simple Summary

Many unique plant species native to the Arabian Peninsula are increasingly threatened by habitat loss and climatic change. Among these are members of the genus *Desmidorchis*, succulent plants traditionally valued for their medicinal importance. This study evaluated the extinction risk of three endemic species and characterized their complete chloroplast genomes to better understand their evolutionary history and genomic diversity. The results classified all three species as Near Threatened (NT), indicating that they may become more vulnerable without effective conservation measures. Comparative plastome analyses revealed a highly conserved genome structure, although several regions, particularly *ycf1* and *clpP1*, showed comparatively elevated sequence divergence and a useful phylogenetic signal. Codon usage and selection-pressure analyses further demonstrated that most plastid genes remain under strong purifying selection, reflecting overall genomic conservation within Stapeliinae. Molecular dating suggested that Arabian *Desmidorchis* diversified relatively recently during the Pleistocene, likely in association with climatic fluctuations and habitat fragmentation in Arabian mountain systems. These findings provide important baseline data for future taxonomic, evolutionary, and conservation studies of rare Arabian succulent plants.

## 1. Introduction

The Arabian Peninsula remains a critical frontier for botanical research and conservation. The high levels of endemism found in its mountain refugia and isolated archipelagos reflect a deep evolutionary history of adaptation to aridity and isolation [1,2]. However, the convergence of climate change, overgrazing, and invasive species poses an unprecedented threat to this unique flora [1]. Central to these conservation concerns are the region’s succulent lineages, which contribute disproportionately to regional endemism and exemplify the evolutionary specialization required to persist in such xeric environments.

The subfamily Asclepiadoideae (Apocynaceae) represents one of the most species-rich and highly endemic groups in the Arabian Peninsula, with endemism reaching approximately 53%, largely driven by leafless stem-succulent stapeliad genera [2]. Within this group, the genus *Desmidorchis* (tribe Ceropegieae, subtribe Stapeliinae) [3] constitutes a taxonomically significant component of the regional succulent flora. The genus comprises approximately fourteen species distributed from tropical East Africa and the Sahara–Sahel region (from Mauritania to Ethiopia) northward into southern Arabia and Socotra [4,5]. Within this broader range, the Arabian Peninsula—particularly Oman and Yemen—serves as a major center of diversity and endemism. According to Ghazanfar [6] and Plants of the World Online (POWO) [4], eight *Desmidorchis* taxa are restricted to the Arabian Peninsula and are not known to occur elsewhere. Five species (*D. adenensis*, *D. aucheriana*, *D. awdeliana*, *D. flava*, and *D. lavrani*) are confined to Oman and Yemen, two species (*D. impostor* and *D. tardellii*) are restricted to Oman, and *D. arabica* is distributed across Oman, Saudi Arabia, the United Arab Emirates, and Yemen. Among these taxa, *D. adenensis*, *D. arabica*, and *D. awdeliana* are particularly notable due to their reported use in traditional medicine [7].

*Desmidorchis adenensis* are used as anti-diabetic, anti-ulcer, anti-inflammatory, anti-parasitic and anti-pyretic agents [8]. *D. arabica* is used as an emollient for sunburns and itchy skin, and tea prepared from the stem is taken for liver problems and painful, inflammatory conditions, while juice of the stem is added to milk to curdle it and given to the sick for convalescence, and as decoction for the treatment of many disease conditions [6,9,10,11]. Similarly, raw pieces of *D. awdeliana* are used to treat diabetes and snake bites [11]. Phytochemical investigations have confirmed the presence of bioactive compounds in these species; for instance, *D. arabica* has yielded six pregnane glycoside, four of them new (Arabincoside A, B, C, and D), while *D. awdeliana* has yielded four pregnane glycosides and a flavone glycoside, including the newly discovered awdeliosides A and B [10,12]. These significant medicinal and economic values underscore the critical importance of sustainable utilization and conservation of their wild resources.

**Figure 1:**
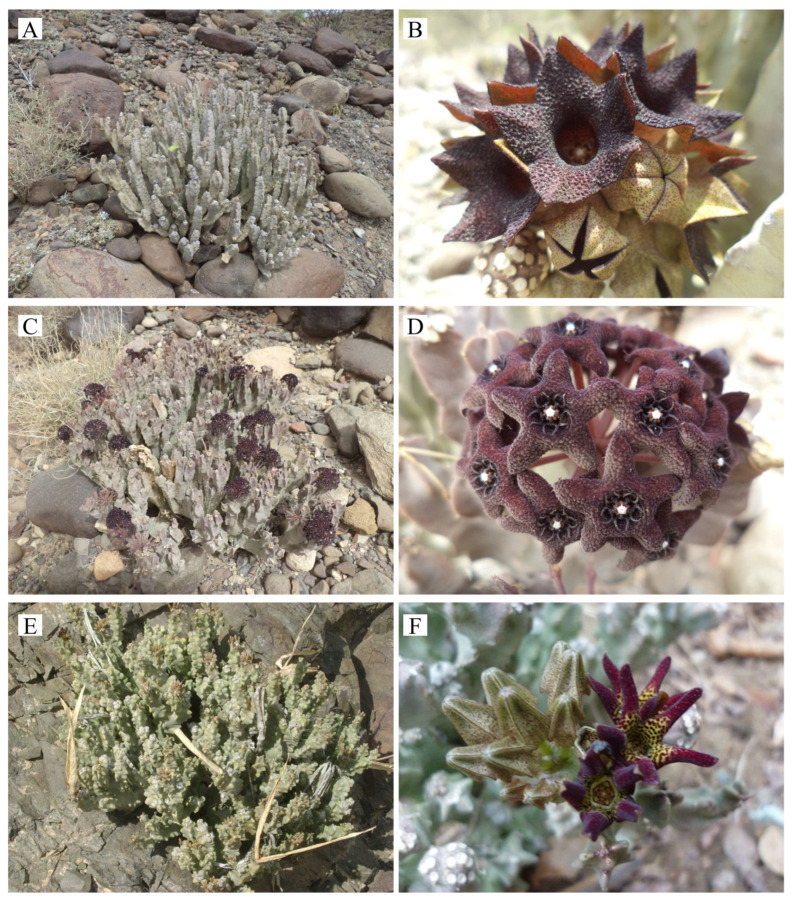
*Desmidorchis adenensis* (**A**,**B**), *D. arabica* (**C**,**D**), and *D. awdeliana* (**E**,**F**) in their natural habitat in the Aqan region, Lahij Governorate, southern Yemen. Photo by O. Al-Hawshabi.

Physically, these three species are succulent perennial herbs, reaching heights of 15–35 cm. They feature stems branched from the base and distinctively angled, terminating in apical umbel-like inflorescences (Figure 1A,C,E). While they share a similar growth habit, they are distinguished by their floral morphology: *D. adenensis* features dark red campanulate flowers with clavate hairs at the lobe tips (Figure 1B); *D. arabica* displays dark purple–red, rotate to shallowly campanulate flowers (Figure 1D); and *D. awdeliana* is characterized by yellow rotate flowers with dark purple spots on the inner surface (Figure 1F) [6,13,14,15]. The specialized succulent morphology and niche habitat requirements of these species, while enabling survival in xeric conditions, render them particularly susceptible to external disturbances [16].

Despite their pronounced physiological resilience and medicinal value, these species are subject to severe anthropogenic and environmental pressures. The most significant threats include destructive harvesting through total uprooting for traditional medicinal use, habitat degradation associated with road construction and infrastructure expansion, and prolonged drought episodes linked to regional climate variability [6,17,18]. Reflecting these pressures, conservation assessments vary markedly at the national level. In Oman, *D. adenensis* is classified as Endangered and *D. arabica* as Vulnerable [6], whereas in Yemen, all three species are considered Near Threatened [19]. In contrast, *D. arabica* is regarded as Least Concern in the United Arab Emirates due to its scattered mountain distribution, although it remains under-recorded and locally rare [20].

Critically, none of these taxa have yet been evaluated under a formal global assessment framework, leaving their worldwide extinction risk unresolved. Establishing a unified global IUCN Red List assessment is therefore essential for these Arabian endemics to ensure consistency across national boundaries and to facilitate coordinated conservation action. Although national red lists play a vital role in shaping local policy [21], they may yield divergent or even misleading conclusions for transboundary species when not integrated within a standardized global framework. A global Red List assessment represents the benchmark for conservation prioritization—often described as a “gold standard” for biodiversity data—guiding funding agencies, international agreements, and recovery planning. Transitioning to a global assessment would also enable more accurate monitoring of population trends through indicators such as the Red List Index, while providing the international recognition necessary to safeguard these unique Arabian lineages from further decline [22].

Genomic sequencing has become a fundamental tool in modern conservation biology, providing high-resolution insights into genetic diversity that are essential for assessing a species’ evolutionary potential and long-term resilience [23,24]. Molecular techniques are increasingly important for the conservation of endemic Arabian flora because they provide precise genetic information that complements traditional morphological approaches. Among available molecular resources, the chloroplast genome has emerged as a particularly valuable tool in plant evolutionary biology owing to its relatively small size, conserved gene content, and generally stable genomic structure [25,26,27]. The usefulness of plastomes for evolutionary studies is closely linked to their predictable patterns of inheritance and structural conservation. Unlike nuclear genomes, which often exhibit extensive recombination and large variation in genome size, chloroplast genomes maintain a highly conserved gene order and content across most angiosperms. This structural stability enables reliable comparisons of homologous regions even among distantly related taxa [28,29]. 

In addition, the predominantly uniparental inheritance of plastids, the absence of meiotic recombination, and relatively slow nucleotide substitution rates make plastomes particularly suitable for reconstructing evolutionary relationships across multiple temporal scales [30,31,32]. When integrated with fossil calibrations or known geological events, plastome data can also be used to estimate divergence times, thereby providing a temporal framework for understanding how aridity and complex geological histories have shaped lineage diversification. Despite this overall stability, plastome evolution is not entirely static. Structural changes such as gene loss or transfer, gene duplication, sequence inversions, and expansion or contraction of inverted repeat (IR) regions are frequently observed in plant plastomes [27,33,34,35]. Comparative analyses of genome size, gene content, IR boundary variation, repetitive sequences, and structural rearrangements therefore provide valuable phylogenetic signals and contribute to a deeper understanding of plastome evolution. 

There has been growing interest in the genomic analysis of Arabian plants during the last decade [36]; however, the genus *Desmidorchis* remains largely underrepresented in plastome studies. The first complete chloroplast genomes in this genus were reported by Alharbi and Albokhari [37], focusing on the two most widely distributed species in the genus: *D. penicillata* and *D. retrospiciens*. More broadly, despite the extensive diversity of Apocynaceae (approximately 5290 species) and the recognized medicinal importance of many of its members [38,39], only about 5% of species currently have publicly available chloroplast genome data in the NCBI database (https://www.ncbi.nlm.nih.gov/). Existing plastome datasets have contributed to resolving broad phylogenetic relationships within the family [40], yet substantial gaps remain in understanding plastome evolution, sequence divergence, and evolutionary dynamics within Stapeliinae.

Accordingly, this study aims to (i) conduct preliminary global-scale conservation assessments of *D. adenensis*, *D. arabica*, and *D. awdeliana* following IUCN Red List criteria; (ii) characterize and compare their complete chloroplast genomes with representative Stapeliinae plastomes; (iii) investigate repeat sequence variation, divergence hotspots, IR shifts, codon usage bias, and selection pressure; (iv) reconstruct phylogenomic relationships within tribe Ceropegieae using plastid protein-coding genes; and (v) estimate divergence times to better understand the evolutionary radiation of Arabian *Desmidorchis* within the context of regional paleoclimatic history.

Together, these analyses aim to improve current genomic and conservation knowledge of Arabian *Desmidorchis* endemics. By integrating comparative plastome genomics with standardized IUCN assessment frameworks, this study provides a useful foundation for future taxonomic, evolutionary, and conservation research on rare Arabian succulent plants.

## 2. Materials and Methods

### 2.1. IUCN Red List Conservation Assessment

The conservation status of *Desmidorchis adenensis*, *D. arabica*, and *D. awdeliana* was evaluated following the Guidelines for Using the IUCN Red List Categories and Criteria Version 16 [41]. In the absence of comprehensive population size estimates and long-term trend data—which are currently unavailable for these taxa due to the remote and rugged nature of their habitats—the assessment was conducted under Criterion B (Geographic Range). The IUCN specifically provides Criterion B as a robust alternative for evaluating extinction risk when demographic data are limited, provided there is evidence of restricted range and a continuing decline [41].

Two standard IUCN metrics were utilized: Extent of Occurrence (EOO), defined as the area contained within the shortest continuous imaginative boundary encompassing all known or inferred sites of a species, and Area of Occupancy (AOO), representing the area actually occupied by the species, calculated using the standard 2 km × 2 km grid cells recommended by the IUCN [41,42]. Both metrics were calculated using the Geospatial Conservation Assessment Tool (GeoCAT, beta version) [43].

To support these assessments, point distribution data, habitat conditions, and identified threats were synthesized from three primary sources: direct field observations in Yemen, an extensive review of specialized scientific literature [6,11,13,14,15,17,18,19,20,44,45,46,47,48,49,50,51,52], and occurrence records from the Global Biodiversity Information Facility (GBIF: https://www.gbif.org/; accessed on 3 July 2025). The occurrence map of the studied species was generated using ArcGIS Online (Esri, Redlands, CA, USA) using the “Topographic” basemap [53]. By utilizing a combination of range-based metrics and documented anthropogenic pressures, this methodology ensures a precautionary and scientifically grounded assessment consistent with international conservation standards.

### 2.2. Chloroplast Genome Analysis

#### 2.2.1. Plant Sampling

Samples of *Desmidorchis adenensis*, *D. arabica*, and *D. awdeliana* were collected in October 2024 from the Aqan region, Al-Musemier District, Lahij Governorate, southern Yemen (13°35′19.0″ N, 44°39′50.0″ E), at an elevation of approximately 520–557 m. For each species, a single individual was sampled. The collection site is not located within a protected area; therefore, no specific collection permits were required. Voucher specimens were prepared by preserving fresh plant material in 70% ethanol and subsequently deposited in the Umm Al-Qura University Herbarium (UQUH).

#### 2.2.2. DNA Extraction, Library Preparation, and Sequencing

Powdered stem tissues derived from the dried specimens of *D. adenensis*, *D. arabica*, and *D. awdeliana* were submitted to Novogene Co. Ltd. (Beijing, China) for total genomic DNA extraction and high-throughput sequencing. DNA was isolated using the FastDNA™ SPIN Kit (MP Biomedicals, Irvine, CA, USA), in accordance with the manufacturer’s instructions. The quality of the extracted DNA was verified through agarose gel electrophoresis, and concentrations were determined using a Qubit Fluorometer (Thermo Fisher Scientific, Waltham, MA, USA).

For library construction, the genomic DNA was fragmented into ~350 bp segments using mechanical shearing. The resulting fragments were processed through end-repair, adenylation at the 3′ ends, and adapter ligation using the Rapid Plus DNA Library Prep Kit for Illumina (ABclonal, Wuhan, China). Subsequent steps included size selection, PCR amplification, and purification using the AMPure XP beads (Beckman Coulter, Beverly, MA, USA). Library integrity was assessed using an Agilent Fragment Analyzer (Agilent Technologies, Santa Clara, CA, USA), and concentrations were measured by Qubit and qPCR.

Sequencing was carried out on the Illumina NovaSeq platform (Illumina Inc., San Diego, CA, USA), generating 150 bp paired-end reads. To ensure high-quality data, raw sequences were processed with Fastp v0.23.1 [54]. The filtering criteria involved removal of (1) reads containing >10 nt of adapter sequences with ≤10% mismatches, (2) reads with more than 10% ambiguous nucleotides (N), and (3) reads where over half the bases had a Phred quality score below 5. This filtering yielded high-quality clean data (Q30 > 91% and an error rate of 0.03%) totaling approximately 4.0–4.2 GB per sample (representing >99% effective reads). Detailed sequencing metrics are provided in Table S1.

#### 2.2.3. Genome Assembly and Annotation

Chloroplast genome assembly was performed using NOVOPlasty v4.3.5 [55], with the plastome of *Desmidorchis penicillata* (GenBank accession: PP101941) serving as the reference. A k-mer values optimized for each species (k = 39 for *D. adenensis*; k = 33 for *D. arabica* and *D. awdeliana*), and the *trnK*-UUU gene, extracted from the reference, was used as the seed sequence. The initial assembly yielded multiple contigs with high average organelle coverage, ranging from 592× to 658×. These contigs were subsequently merged into a complete circular chloroplast genome using the de novo assembly feature in Geneious Prime^®^ v2025.0.3 [56]. To ensure structural integrity, the clean reads were mapped back to the finalized consensus sequences to verify coverage uniformity and the accuracy of the Inverted Repeat (IR) junctions.

Automated genome annotation was carried out using GeSeq v2.03 [57], followed by manual curation and refinement within Geneious Prime to ensure correct start/stop codons and exon–intron boundaries. The sizes and boundaries of the IR regions were verified using the “Find Repeats” tool in Geneious Prime. Structural validation of the annotated plastomes was conducted using GB2sequin v1.0 [58]. The finalized sequences were based on high-quality data (Q30 > 91%) and have been submitted to GenBank under accession numbers PV946908, PV946909, and PV946910 for *D. adenensis*, *D. arabica* and *D. awdeliana*, respectively.

#### 2.2.4. Characterization of Chloroplast Genomes

Genome characteristics—including total length, gene content, intron–exon organization, and GC content—were analyzed using Geneious Prime. Circular representations of the chloroplast genomes were generated using OrganellarGenomeDRAW v1.3.1 [59].

#### 2.2.5. Comparative Genomic Analysis Dataset

For the analysis of sequence repeats, hypervariable regions, IR expansion and contraction, codon usage bias, and selection pressure analysis, the three newly sequenced chloroplast genomes of *Desmidorchis* species were compared with twelve previously published complete plastomes from other Stapeliinae taxa available in GenBank. Those taxa are *Ceropegia dolichophylla* (OP133580), *Ceropegia longifolia* (OP133586), *Ceropegia nilotica* (OP133584), *Ceropegia sunhangiana* (OR260539), *Desmidorchis penicillata* (PP101941), *Desmidorchis retrospiciens* (PP291741), *Duvalia velutina* (MT431578), *Huernia keniensis* (OP133582), *Monolluma quadrangula* (MT413385), *Orbea variegata* (NC_079601), *Orbea sprengeri* subsp. *commutata* (PQ412530) (hereafter *Orbea sprengeri*), and *Orbea wissmannii* var. *eremastrum* (PQ412531) (hereafter *Orbea wissmannii*).

#### 2.2.6. Repetitive Sequence Analysis

Simple sequence repeats (SSRs), dispersed repeats (forward, reverse, palindromic, and complementary) and long tandem repeats (repeat unit size ≥ 7 bp) were identified in the aforementioned fifteen Stapeliinae plastomes.

SSRs were identified using the Perl script MISA (MIcroSAtellite) v2.1 [60]. Detection parameters were set to a minimum threshold of 10, 5, 4, 3, 3, and 3 repeat units for mono-, di-, tri-, tetra-, penta-, and hexanucleotides, respectively. Following a conservative approach to ensure the independence of each locus, the minimum distance between adjacent SSRs was defined as 0 bp. This setting ensured that all SSRs were recorded as individual units rather than being merged into compound sequences, providing a more granular assessment of repeat distribution across the plastomes.

Two key metrics were calculated to characterize SSR distribution patterns following Zhu et. al., [61]: relative abundance and relative density. Relative abundance was defined as the number of SSRs per kilobase of genome sequence (SSRs/kb), and relative density was defined as the total length of SSRs (bp) per kilobase of genome sequence (bp/kb).

To further examine lineage-associated SSR motifs, the coordinate files generated in the ‘*.fas.misa*’ format from all fifteen plastomes were imported into Geneious Prime and linked to their corresponding plastome sequences for comparative inspection of shared and potentially distinctive motif patterns among the analyzed taxa.

Dispersed repeats were identified using the REPuter program [62] with default settings, specifying a maximum computed repeats of 50 and minimum repeat size of 8 bp. Tandem repeats were detected using Tandem Repeats Finder [63] with default parameters: match score = 2, mismatch = 7, indel = 7, minimum alignment score = 50, and maximum period size = 500.

#### 2.2.7. Identifying Hypervariable Regions

Nucleotide diversity (π) across the fifteen chloroplast genomes was analyzed using DnaSP v6.12.03 [64] with a sliding window strategy, applying a window size of 800 bp and a step size of 200 bp. Sequence divergence and highly variable regions were further assessed using the mVISTA program [65], with genome alignments performed in Shuffle-LAGAN mode using the *Desmidorchis retrospiciens* chloroplast genome as a reference. This genome was selected as a well-annotated and complete reference for the genus.

Hypervariable regions identified through sliding-window analysis and mVISTA comparisons were subsequently evaluated for their phylogenetic informativeness using the approaches described in Section 2.3 (Phylogenomic Analysis). Regions showing the strongest phylogenetic resolution were further subjected to BLASTn analyses using the NCBI Basic Local Alignment Search Tool [66] to assess their discriminatory potential among *Desmidorchis* and related Stapeliinae taxa.

#### 2.2.8. IR Boundary Comparison

To examine the structural organization and variation at the junctions of the large single-copy (LSC), small single-copy (SSC), and inverted repeat (IR) regions, the boundaries of all fifteen Stapeliinae chloroplast genomes were analyzed using IRscope [67]. The chloroplast genome of *Desmidorchis retrospiciens* was used as the reference for alignment and comparative assessment.

#### 2.2.9. Codon Usage Bias Analysis

Relative synonymous codon usage (RSCU) was applied to assess codon usage bias by comparing the observed frequency of each synonymous codon with its expected frequency under equal usage [68]. Protein-coding sequences (CDS) were retrieved from annotated chloroplast genomes using Geneious Prime, and sequences that were duplicated, incomplete, or truncated were excluded prior to analysis. RSCU values were calculated using MEGA X [69], and codon usage patterns were interpreted such that RSCU values greater than one indicate preferential usage, values less than one indicate under-representation, and values equal to one suggest no bias. This analysis was performed using 79 of the 80 plastid protein-coding genes, with *ycf15* excluded because of its absence in several analyzed plastomes, to provide a comprehensive overview of codon usage across the plastome.

To further investigate the factors shaping codon usage bias, additional analyses were conducted based on a filtered dataset in which coding sequences shorter than 300 bp were excluded to reduce statistical bias, resulting in a total of 53 genes. The effective number of codons (ENC) was calculated using CodonW v1.4.2 [70], and a scatter plot was constructed by plotting ENC values against GC content at the third codon position (GC3) to visualize codon usage patterns among genes and to assess the relative contributions of mutational pressure and natural selection to codon usage bias [71]. A standard curve representing the expected ENC values under mutational bias alone was calculated and overlaid on the plot. Genes distributed close to this curve were considered to be mainly influenced by mutational pressure, whereas genes deviating from the expected distribution were interpreted as being affected by additional selective forces.

In addition, parity rule 2 (PR2) plot analysis was carried out to examine the mutational equilibrium at the third codon position [72]. A two-dimensional scatter plot was generated using G3/(G3 + C3) as the horizontal axis and A3/(A3 + T3) as the vertical axis. Reference lines were drawn at 0.5 on both axes to indicate equal usage of complementary bases (G versus C and A versus T). The position of genes relative to the center point (0.5, 0.5) was used to infer the extent of deviation from compositional equilibrium, thereby reflecting the combined influence of mutation and natural selection on codon usage patterns. Data visualization and calculations were performed using Microsoft Excel [73].

#### 2.2.10. Selection Pressure Analysis

Selective pressure acting on 79 shared chloroplast protein-coding genes was evaluated across 15 Stapeliinae plastomes using pairwise Ka/Ks comparisons. Coding sequences (CDS) for each orthologous gene were aligned in Geneious Prime, and nonsynonymous (Ka) and synonymous (Ks) substitution rates were calculated in TBtools-II [74] using the Nei–Gojobori (NG) method. Pairwise Ka/Ks ratios were estimated among the examined taxa to assess patterns of selective pressure across plastid genes. A Ka/Ks ratio greater than 1 was interpreted as evidence of positive or accelerated selection, a ratio equal to 1 indicated neutral evolution, and a ratio below 1 reflected purifying selection. Heatmap visualization of pairwise Ka/Ks values was generated using TBtools-II.

### 2.3. Phylogenomic Analysis

Phylogenomic relationships of Arabian *Desmidorchis* species were reconstructed using a dataset comprising 24 chloroplast genomes, including 21 ingroup taxa and 3 outgroup species from tribe Marsdenieae: *Hoya exilis* (MW719054.1), *Hoya megalaster* (MW719063.1), and *Hoya ariadna* (OL754671.1). These outgroups were selected because Marsdenieae is consistently recovered as the sister lineage to tribe Ceropegieae in previous studies [39,40], ensuring accurate rooting of the phylogenetic tree. The ingroup included fifteen Stapeliinae plastomes (see Section 2.2.5), supplemented by six additional Ceropegieae species retrieved from GenBank.: *Sisyranthus trichostomus* (KF539852; subtribe Anisotominae, partial plastome), *Heterostemma oblongifolium* (OP133585; subtribe Heterostemminae), *Pentasachme caudatum* (MW136321; subtribe Leptadeniinae), *Leptadenia albida* (MG963261; subtribe Leptadeniinae, partial plastome), *Leptadenia pyrotechnica* (MW546533; subtribe Leptadeniinae), and *Stapelia gigantea* (MG963259), a partial plastome from subtribe Stapeliinae.

Phylogenomic analyses were based on a concatenated dataset of 80 chloroplast protein-coding sequences (CDS), with missing data permitted for genes absent in some plastomes. Each gene was aligned individually using MUSCLE v3.8.4 [75] as implemented in Geneious Prime with default parameters, and the resulting alignments were concatenated into a supermatrix. Maximum likelihood (ML) analysis was performed in RAxML-HPC BlackBox v8.2.12 [76] with 1000 bootstrap replicates, and Bayesian inference (BI) was conducted in MrBayes v3.2.7 [77,78] using two independent runs. Both analyses were carried out under a partitioned scheme, with partitions defined according to the best-fit partitioning strategy identified using PartitionFinder v2.1.1 [79] under the corrected Akaike Information Criterion (AICc). All phylogenetic analyses were executed via the CIPRES Science Gateway [80].

Markov chain Monte Carlo (MCMC) analyses were run for 10 million generations, with sampling every 10,000 generations. Convergence was assessed using Tracer v1.7.1 [81], with effective sample sizes (ESS) greater than 200 considered indicative of convergence. The first 25% of sampled trees were discarded as burn-in, and a majority-rule consensus tree was generated from the remaining trees. Tree visualization was performed in iTOL v7.2.1 [82].

To assess the phylogenetic utility of hypervariable regions identified through sliding-window analysis and mVISTA, maximum likelihood analyses with 1000 bootstrap replicates were performed on the full dataset of 24 plastomes, comprising 21 Ceropegieae species and 3 outgroup taxa. The most variable regions—*ycf1*, *clpP1*, *ycf1-trnN*, and *psbM-trnD*—were tested independently as well as in seven combinations (*ycf1* + *clpP1*, *clpP1* + *ycf1-trnN*, *psbM-trnD* + *ycf1-trnN*, *ycf1* + *psbM-trnD*, *ycf1* + *ycf1-trnN*, *clpP1* + *psbM-trnD*, and *ycf1* + *clpP1* + *psbM-trnD* + *ycf1-trnN*).

### 2.4. Divergence Time Estimation

Divergence times were estimated using a Bayesian relaxed molecular clock approach. To incorporate available fossil evidence for subfamily Asclepiadoideae, taxon sampling was expanded to include representatives of all tribes and subtribes within the subfamily, as well as available plastomes of subfamily Secamonoideae, resulting in a total of 54 taxa (Table S2). The analysis was based on a concatenated dataset of 62 shared CDS, which were aligned individually using MUSCLE and concatenated into a supermatrix following removal of ambiguously aligned regions. To accommodate substitutional heterogeneity, the optimal partitioning scheme and substitution models were determined using PartitionFinder v2.1.1 under AICc.

Bayesian divergence time analyses were conducted in BEAST v2.7.8 [83] via the CIPRES Science Gateway with input files generated in BEAUti v2.7.8. An uncorrelated lognormal relaxed clock model was applied, and a Yule speciation process was used as the tree prior. Two fossil-based calibration points were implemented using lognormal prior distributions. The first calibration was applied to the crown node of Asclepiadoideae based on fossil evidence (*Asclepiadospermum*, early Eocene) [84], with an offset of 47.8 Ma, a mean (M) of 0.8, and a standard deviation (S) of 0.7. The second calibration was applied to the stem node of subtribe Tylophorinae based on the fossil *Tylophora antiqua* (early–middle Oligocene) [85], with an offset of 28 Ma, a mean (M) of 0.8, and a standard deviation (S) of 0.7. The calibrated node corresponded to the divergence between the sampled Tylophorinae clade and its sister lineage, subtribe Asclepiadinae.

The MCMC analyses were run for 100 million generations, with sampling every 10,000 generations. Two independent runs were conducted to ensure convergence. Log files were examined in Tracer v1.7.1, and ESS values greater than 200 were obtained for all parameters. The first 25% of samples were discarded as burn-in. Log and tree files were combined using LogCombiner v2.7.9, and a maximum clade credibility (MCC) tree with mean node heights and 95% highest posterior density (HPD) intervals was generated using TreeAnnotator v2.7.9. The resulting chronogram was visualized in FigTree v1.4.4 [86]. The tree was plotted against stratigraphy using the strap package [87] in RStudio v4.6.0 [88].

## 3. Results

### 3.1. Conservation Status and Distribution Patterns

**Figure 2:**
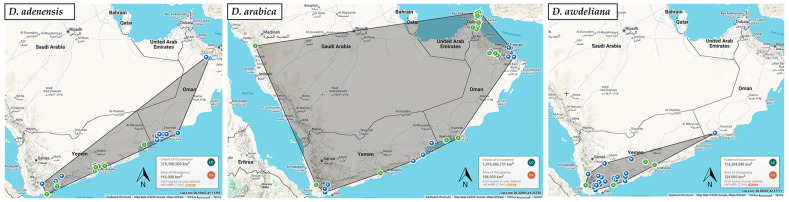
Distribution and range assessments of *Desmidorchis adenensis*, *D. arabica*, and *D. awdeliana* in the Arabian Peninsula generated using GeoCAT. The maps display the Extent of Occurrence (EOO; shaded polygon) and Area of Occupancy (AOO; calculated using a 2 km × 2 km grid cell). Green circles represent occurrence points derived from GBIF, while blue circles indicate data obtained from field observations and literature review.

All three Arabian endemics—*Desmidorchis adenensis*, *D. arabica*, and *D. awdeliana*—were assessed in this study as Near Threatened (NT) at the global scale. While their Extent of Occurrence (EOO) exceeds the thresholds for threatened categories (ranging from 132,285 km^2^ for *D. awdeliana* to over 1.87 million km^2^ for *D. arabica*; Figure 2), their Areas of Occupancy (AOO) are considerably restricted (116 km^2^ for *D. adenensis*, 184 km^2^ for *D. arabica*, and 124 km^2^ for *D. awdeliana*). These AOO values fall within the Endangered (EN < 500 km^2^) range under Criterion B2. However, the taxa do not currently satisfy the full set of required subcriteria (at least two of three) for inclusion within a threatened category. Specifically, there is no evidence of severe fragmentation (B2a) or extreme fluctuations (B2c). Nevertheless, the Near Threatened (NT) designation is supported by inferred continuing declines in habitat quality and AOO driven by synergistic anthropogenic and environmental pressures under Criterion B2b (ii,iii), indicating that the species are close to qualifying for the Endangered category.

**Figure 3:**
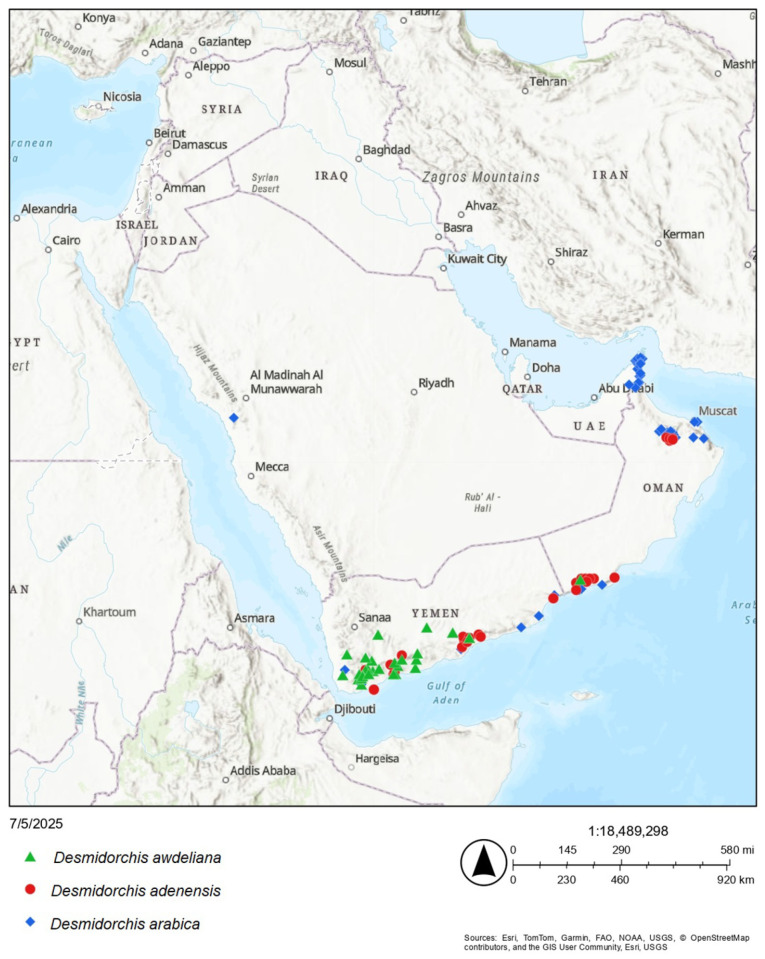
Occurrence map of *Desmidorchis adenensis*, *D. arabica*, and *D. awdeliana* in the Arabian Peninsula. Created by ArcGIS Online (Esri, “Topography”) [53].

Ecologically, these species are well adapted to xeric environments, typically inhabiting well-drained rocky substrates, limestone cliffs, and mountain escarpments that act as bioclimatic refugia [6,49] (Figure 3). *Desmidorchis adenensis* is largely confined to the escarpment hills of Dhofar and the rugged terrains of Yemen (18–700 m elevation), often occurring in association with *Adenium* and *Commiphora* [6,49]. *Desmidorchis arabica* exhibits a broader but fragmented distribution, ranging from sea level to approximately 2300 m across the Hajar Mountains of northern Oman and the United Arab Emirates, extending into Yemen and reaching a small, isolated population at Jabal Waggas in Saudi Arabia [6,14,46,49]. In contrast, *D. awdeliana* is primarily centered in Yemen in drained rocky outcrops, with a marginal and potentially vulnerable presence along the drier wadi systems of southern Oman at around 350 m elevation [6,44,49,51].

These habitats are increasingly compromised by several pressures. National-level data underscore this vulnerability: in Oman, the narrow geographic range and habitat degradation from track-road construction and flash flooding have led to a Vulnerable status for *D. arabica* and Endangered classifications for *D. adenensis* [6]. In Yemen, field observations—particularly in the Sultanate of Awdali (the type locality for *D. awdeliana*)—indicate that medicinal overharvesting and use as a food resource are critical drivers of decline; plants are frequently uprooted entirely, preventing regeneration. Furthermore, *Desmidorchis* species often inhabit volcanic rocks with low moisture retention, and prolonged dry seasons can lead to complete desiccation with limited regrowth. Although *D. arabica* is relatively widespread in the UAE, the combined effects of climate change on high-elevation Hajar populations and infrastructure expansion near lowland habitats suggest that, without intervention, these populations may face local extinction [17,20,46]. While AOO estimates derived from opportunistic records are sensitive to sampling effort, the combination of slow growth rates, habitat specificity, and increasing infrastructure development suggests that these taxa are likely to qualify for a threatened category in the near future if current trends persist.

### 3.2. Plastome Structure and Comparative Features

#### 3.2.1. General Plastome Features

**Figure 4:**
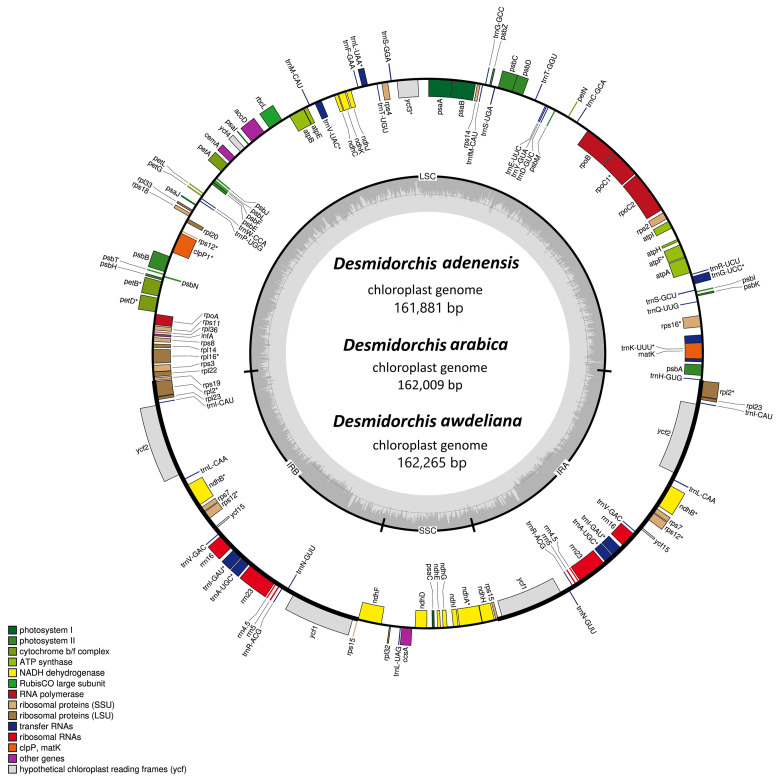
Circular map of the chloroplast genomes of *Desmidorchis adenensis*, *D. arabica*, and *D. awdeliana*. Genes shown on the outside of the circle are transcribed counterclockwise, while those on the inside are transcribed clockwise. The inner gray circle represents GC content variation. Gene functional categories are color-coded. An asterisk (*) indicates genes containing introns.

The complete chloroplast genomes of *Desmidorchis adenensis*, *D. arabica*, and *D. awdeliana* exhibit a typical quadripartite structure, comprising a large single-copy (LSC) region, a small single-copy (SSC) region, and two inverted repeats (IRs) (Figure 4). The total genome sizes are highly similar, ranging from 161,881 bp in *D. adenensis* to 162,265 bp in *D. awdeliana* (Table 1). These values are consistent with those reported for *D. penicillata* (161,776 bp) and *D. retrospiciens* (161,861 bp), indicating overall structural conservation across the genus (Table 1).

The lengths of the LSC, SSC, and IR regions vary only slightly among the species, with the LSC spanning 86,125–86,326 bp, SSC 13,263–13,266 bp, and IRs 31,245–31,338 bp. Coding regions represent approximately 52.6–52.7% of the plastome, with the remainder consisting of non-coding sequences (47.2–47.3%). The overall GC content is nearly uniform, with values ranging between 37.7% and 37.8%, while the AT content reaches up to 62.3%. The GC content varies slightly across the plastome regions: the IR regions exhibit the highest GC content (40.9–41.0%), followed by the LSC (36.2%), and the SSC (32.8%). These values are nearly identical to those recorded in *D. penicillata* and *D. retrospiciens* (Table 1), further supporting the conserved nature of plastome architecture in *Desmidorchis*.

Circular genome maps of *D. adenensis*, *D. arabica*, and *D. awdeliana* reveal a highly conserved gene arrangement, with genes distributed on both strands (Figure 4). Functional gene categories—including those involved in self-replication (e.g., rRNAs and tRNAs), photosynthesis (e.g., *psa*, *psb*, *pet*, *ndh*), and other essential functions such as transcription (*rpo* genes), translation, and protein processing (*matK*, *clpP*)—are consistently represented in all three species (Table 2).

A total of 133 genes were identified in each plastome, comprising 88 protein-coding genes, 37 tRNA genes, and 8 rRNA genes (Table 2). Several genes contain introns, with a conserved pattern across the species. Genes such as *clpP1*, *rps12*, and *ycf3* harbor two introns, while others including *atpF*, *ndhA*, *rpl16*, and *rpoC1* contain a single intron (Table 3). The *clpP1* gene, for example, exhibits a complex structure with three exons and two introns of variable lengths among the species.

#### 3.2.2. Distribution and Composition of cpSSRs

The characteristics of SSRs—including number, size, motif profile, relative abundance, relative density, GC content, and distribution—in the newly sequenced chloroplast genomes of *Desmidorchis* were compared with those of previously published plastomes of Stapeliinae species to assess intra- and inter-genus variability in SSR. The comparative analysis of these fifteen Stapeliinae plastomes reveals that while the genus *Desmidorchis* maintains a remarkably stable and conserved genomic profile—characterized by nearly uniform SSR abundance (0.46–0.48 No./kb) and a narrow density range (5.56–5.97 bp/kb)—other genera like *Orbea* exhibit significant internal divergence, particularly with *O. wissmannii* acting as a major outlier in both total SSR length (1434 bp) and relative density (8.38 bp/kb). Furthermore, the genus *Ceropegia* shows a distinct trend toward higher SSR saturation and elevated GC content compared to the rest of the subtribe, suggesting that while the overall chloroplast genomes are relatively similar in size (averaging ~161 kb), the distribution, chemical composition, and proliferation of repeat sequences serve as key indicators of evolutionary divergence between these botanical lineages (Table 4).

**Figure 5:**
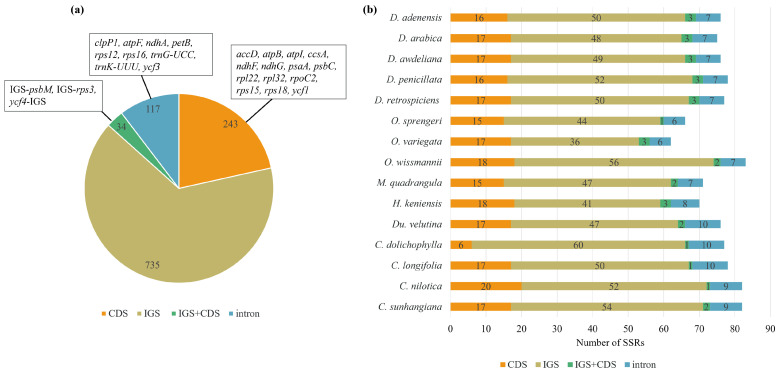
Distribution of SSRs in fifteen Stapeliinae chloroplast genomes. (**a**) Proportions of SSRs in coding sequences (CDS), intergenic spacers (IGS), introns, and CDS-IGS junctions. (**b**) Species-wise SSR counts by genomic feature, showing the dominance of IGS-localized SSRs.

Across all fifteen Stapeliinae plastomes, 1129 SSRs were detected. Most SSRs (735; ~65%) were in intergenic spacers (IGS), followed by coding sequences (CDS) (243; 21.6%), largely in *psaA*, *psbC*, *rpl22*, *rpoC2*, and *ycf1*, and introns (117; 10.4%) in *clpP1*, *atpF*, *ndhA*, *petB*, *rps12*, *rps16*, *trnG-UCC*, *trnK-UUU*, and *ycf3*. A small fraction (34 SSRs; 3.0%) spanned CDS-IGS boundaries, often in IGS-*psbM*, IGS-*rps3*, and *ycf4*-IGS (Figure 5a, Table S3). Within *Desmidorchis*, SSR organization was notably uniform: 48–52 SSRs were in IGS, 16–17 in CDS, 7 in introns, and 3 at CDS–IGS junctions (Figure 5b, Table S3). Other genera showed more variation. *Ceropegia dolichophylla* held the highest IGS SSR count (60), while *C. longifolia*, *Huernia kenienis*, and *Duvalia velutina* each had ten intronic SSRs—the maximum observed. In contrast, *Orbea sprengeri* and *O. wissmannii* carried only one and two CDS-IGS SSRs, respectively (Figure 5b).

**Figure 6:**
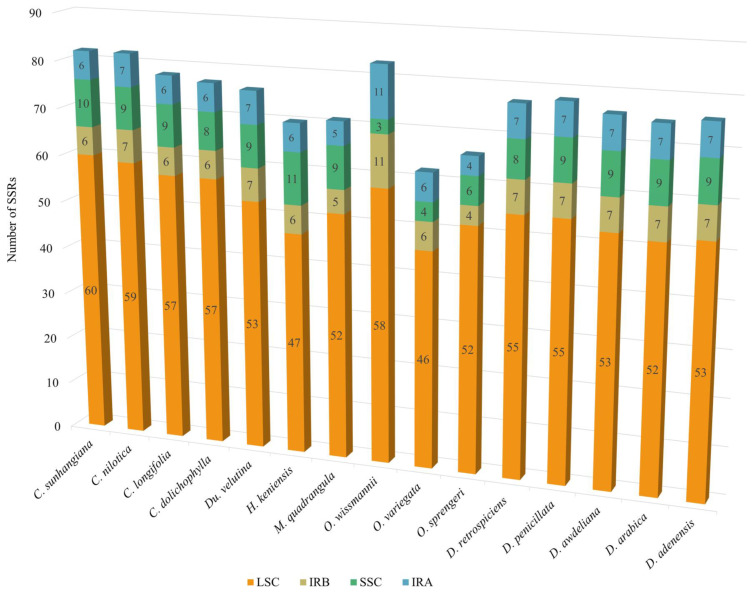
SSR distribution across chloroplast genome regions in fifteen Stapeliinae species. LSC, large single-copy region; SSC, small single-copy region; IRA and IRB, inverted repeat regions.

SSR distribution across the chloroplast compartments (LSC, SSC, IRa, IRb) was uneven (Figure 6, Table S3). The large single-copy (LSC) region consistently held the majority, ranging from 46 SSRs in *O. variegata* to 60 in *C. sunhangiana*. The small single-copy (SSC) region contained 3–11 SSRs, with *O. wissmannii* having the lowest SSC count (3). Inverted repeats (IRs) contained the fewest SSRs (4–7 each) across most taxa, except in *O. wissmannii*, which had 11 per IR, reflecting IR expansion. *Desmidorchis* plastomes displayed the most consistent pattern (52–55 SSRs in LSC, 7 in each IR, 8–9 in SSC). *Ceropegia* species tended to have slightly higher LSC counts and greater SSC/IR variability, while *Orbea* plastomes, particularly *O. wissmannii*, showed distinct compartmental expansions.

**Figure 7:**
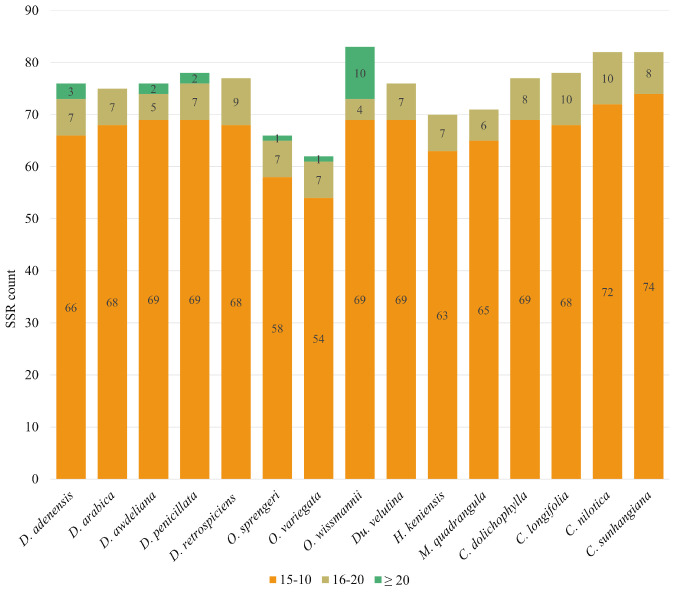
SSR size distribution across the fifteen Stapeliinae plastomes.

The size distribution of SSRs across the fifteen Stapeliinae plastomes is summarized in Figure 7. Most SSRs fell into the 10–15 bp category, reflecting a strong conservation of short repeats across all species. Counts in this size class ranged from 54 in *O. variegata* to 74 in *C. sunhangiana*, with most taxa clustering around 65–72 SSRs. The 16–20 bp class was less common but broadly represented, with counts ranging from only 4 SSRs in *O. wissmannii* to 10 SSRs in *C. longifolia* and *C. nilotica*. Long SSRs (≥20 bp) were rare, few *Desmidorchis* species contained them (3 in *D. adenensis*, 2 each in *D. awdeliana* and *D. penicillata*), and they were entirely absent from *D. arabica* and *D. retrospiciens*. Occasionally long SSRs were present in *O. sprengeri* and *O. variegata* (1 each). In contrast, *O. wissmannii* stood out, carrying 10 SSRs longer than 20 bp, far more than any other species.

**Figure 8:**
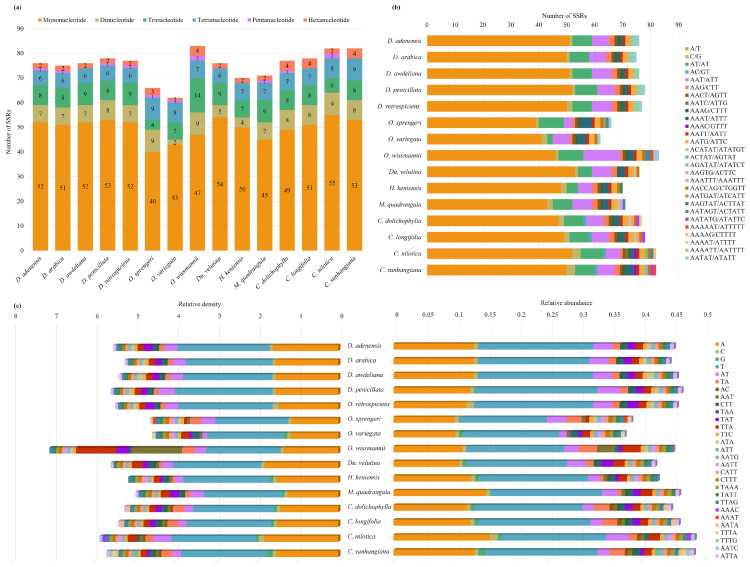
SSR motif composition and distribution across fifteen Stapeliinae plastomes. (**a**) Frequency of SSR motif types by species. (**b**) Total SSR counts by motif type. (**c**) Relative density and abundance of mono- to tetra-SSR motifs per species. Labels on the right indicate the corresponding SSR motif sequences in panels (**b**) and (**c**).

Mononucleotide SSRs dominated all Stapeliinae plastomes, but their abundance and GC content varied across species and genera (Figure 8 and Table 5, Tables S4 and S5). *Desmidorchis* species were remarkably uniform, each carrying 51–53 mononucleotide SSRs with a very low GC content (0.02–0.03), underscoring their conserved A/T-rich motif profile. Trinucleotides were the second most abundant class (e.g., AAT/ATT), ranging from 8 to 9 in *Desmidorchis* to a peak of 14 in *O. wissmannii*, which displayed the greatest motif diversity. Dinucleotides (mostly AT/TA) and tetranucleotides (primarily AATG/ATTC) occurred at moderate levels (6–9 per plastome), showing little variation within *Desmidorchis* but greater variability in *Orbea*. Pentanucleotide and hexanucleotide SSRs were rare across the dataset, yet *O. wissmannii* and *Ceropegia* carried up to four hexanucleotide repeats, whereas *Desmidorchis* plastomes had only one or two for Penta- and hexanucleotide, respectively.

The hexanucleotide AAATTA emerged as a unique *Desmidorchis* marker, consistently located in the *trnN-GUU*–*ycf1* spacer (four copies in *D. penicillata*, *D. awdeliana*, and *D. adenensis*; three in *D. arabica* and *D. retrospiciens*). In addition, the motif ATTA appeared three times in each plastome in the IGS-*psbM* region. Two further motifs—TATT(3) in *trnQ-UUG*–*psbK* and TTA(4) in IGS–*rps3*—were restricted to the stem succulent stapeliads (*Desmidorchis*, *Orbea*, *Huernia*, *Monolluma*, *Duvalia*) and absent in non-succulent *Ceropegia* (Table S3). The number, relative abundance, and relative density of SSRs of different base types in Stapeliinae chloroplast genomes are shown in Figure 8c and Table S4.

#### 3.2.3. Patterns of Dispersed and Tandem Repeats

**Figure 9:**
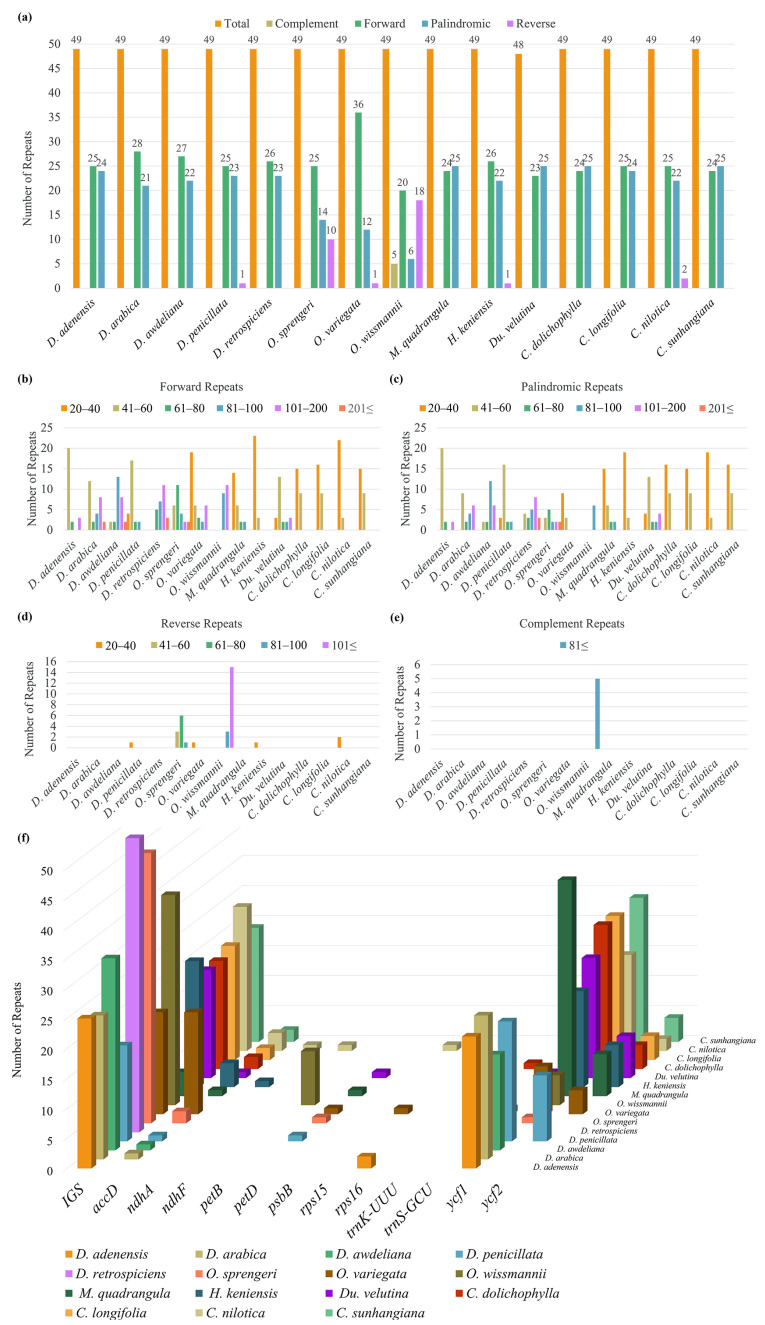
Dispersed repeat sequences in the chloroplast genomes across fifteen Stapeliinae plastomes, showing (**a**) the total number of repeats, their length distribution across (**b**) forward, (**c**) palindromic, (**d**) reverse, and (**e**) complement types, and (**f**) their genomic distribution.

Analysis of dispersed repeats revealed that forward and palindromic types were the most prevalent across Stapeliinae plastomes, whereas reverse and complementary repeats occurred at much lower frequencies (Figure 9a and Table S6). Most forward and palindromic repeats were distributed within the 20–40 bp and 41–60 bp length categories, with only a few species containing longer repeats (>100 bp) (Figure 9b–e). Among *Desmidorchis* species, *D. arabica* exhibited the highest number of forward repeats (28) but the lowest number of palindromic repeats (21). All *Desmidorchis* plastomes contained both forward and palindromic repeats, although *D. penicillata* was unique in also harboring a single reverse repeat.

Gene-level mapping of dispersed repeat locations (Figure 9f) showed that the majority were concentrated in large protein-coding genes, particularly *ycf1* and *ycf2*, as well as in intergenic spacer regions. These loci consistently contained abundant repeats across all species, although the absolute numbers varied among taxa.

**Figure 10:**
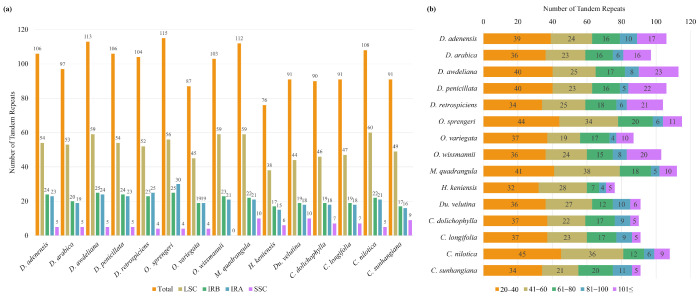
Distribution of tandem repeats across fifteen Stapeliinae plastomes. (**a**) Bar chart showing the number of tandem repeats in each plastome, partitioned by genomic regions: LSC, large single-copy region; SSC, small single-copy region; IRA and IRB, inverted repeat regions. (**b**) Horizontal stacked bars depicting the abundance of tandem repeats by repeat length classes.

Analysis of tandem repeats across Stapeliinae plastomes revealed consistent regional biases (Figure 10a and Table S7). In all species, the LSC region harbored the majority of tandem repeats, followed by the inverted repeat regions (IRa and IRb), while the SSC region consistently contained the fewest. Within *Desmidorchis*, *D. awdeliana* (113 repeats) exhibited the highest number of tandem repeats, whereas *D. arabica* (97 repeats) had the lowest. Among other taxa, *Orbea sprengeri* displayed the greatest overall number of tandem repeats (115), while *O. variegata* contained the fewest (87).

Length-class analysis (Figure 10b and Table S8) showed that most tandem repeats were concentrated in the 20–40 bp category, followed by the 41–60 bp class. Longer repeats (>100 bp) were comparatively rare and occurred only sporadically across species. Notably, *D. awdeliana*, *D. penicillata*, and *D. retrospiciens* harbored higher proportions of long repeats compared with their congeners.

#### 3.2.4. Sequence Divergence and Hypervariable Regions

**Figure 11:**
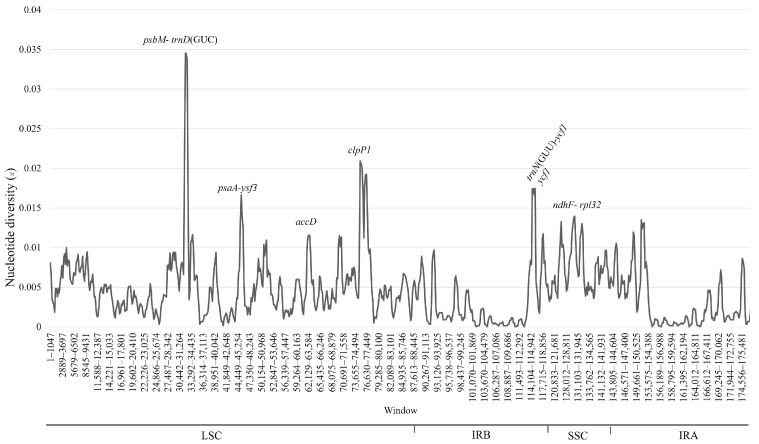
Sliding window analysis of nucleotide diversity (π) among 15 chloroplast genomes of subtribe Stapeliinae. Nucleotide diversity was calculated using a window length of 800 bp with a step size of 200 bp across the aligned plastome sequences. LSC, large single-copy region; SSC, small single-copy region; IRA and IRB, inverted repeat regions.

Hypervariable regions were identified through both sliding window analysis and mVISTA alignment across the fifteen Stapeliinae chloroplast genomes. Both approaches revealed a high level of sequence conservation, particularly within coding regions. The sliding window analysis (Figure 11) showed that most regions exhibited low nucleotide diversity (π < 0.01), indicating strong genomic conservation across taxa. However, several hypervariable regions exhibited elevated π values, with the *psbM–trnD* intergenic spacer showing the highest peak (~0.033), followed by *clpP1*, *trnN-ycf1*, *ycf1*, *psaA-ycf3*, and *ndhF-rpl32*. The IR regions were the most conserved, displaying minimal sequence divergence across all species (Figure 11).

**Figure 12:**
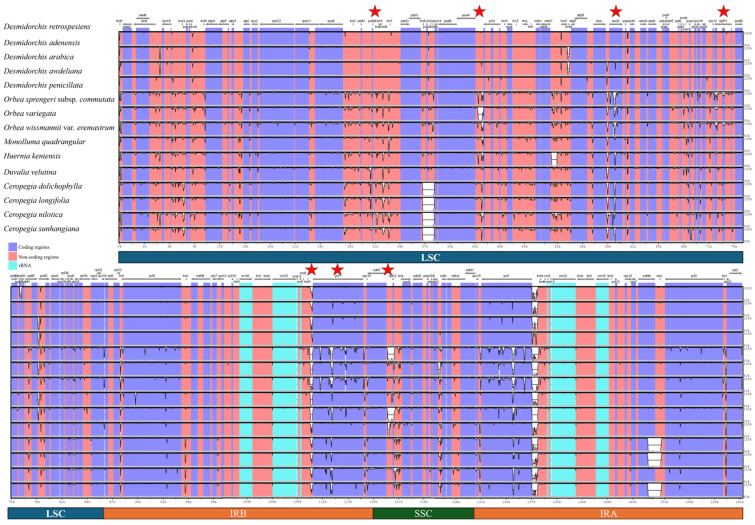
mVISTA alignment of fifteen Stapeliinae chloroplast genomes using *Desmidorchis retrospiciens* as reference. Gray arrows above the alignment indicate gene orientation and transcriptional direction in the reference plastome. Red stars indicate hypervariable regions identified through sliding-window analysis of nucleotide diversity (π). LSC, large single-copy region; SSC, small single-copy region; IRA and IRB, inverted repeat regions.

The mVISTA alignment results were consistent with the sliding window analysis, confirming overall sequence conservation among Stapeliinae plastomes (Figure 12). All five *Desmidorchis* species exhibited nearly identical profiles, with minimal divergence across both coding and non-coding regions, underscoring their genomic stability. By comparison, greater sequence variation was observed in *Orbea* and *Ceropegia*, particularly within non-coding intergenic spacers such as *trnH-psbA*, *psbM-trnD*, *trnN-ycf1*, and *psaA-ycf3*, as well as in variable coding genes including *accD*, *clpP1*, and *ycf1*.

Testing the phylogenetic informativeness of individual hypervariable regions and their combinations was performed using maximum likelihood analyses, and the resulting phylogenetic trees are presented in the Supplementary Materials (Figure S1). Overall, most regions exhibited weak to moderate phylogenetic resolution when analyzed individually. Among the tested combinations, the *ycf1* + *clpP1* dataset showed the strongest phylogenetic performance, successfully resolving the monophyly of *Desmidorchis*, stapeliads, the subtribe Stapeliinae, and the tribe Ceropegieae with high statistical support. The resulting topology was largely congruent with the 80-CDS supermatrix tree (Section 3.3, Phylogenomic Relationships), although minor topological differences were observed within the *Desmidorchis* clade and in the placement of *Ceropegia nilotica* and *Duvalia velutina*.

Further evaluation of the best-performing marker combination (*ycf1* + *clpP1*) using BLASTn analyses demonstrated that all examined *Desmidorchis* accessions consistently recovered other *Desmidorchis* plastomes as the closest matches, with sequence identities exceeding 99% (Table S9). Closely related Stapeliinae genera, particularly *Orbea*, *Ceropegia*, and *Huernia*, exhibited slightly lower sequence identities. Although the combined marker was not strictly genus-specific, these results indicate that the *ycf1* + *clpP1* region retains sufficient sequence divergence to differentiate *Desmidorchis* from related Stapeliinae genera while remaining highly conserved within the genus.

#### 3.2.5. IR Boundary Variation

**Figure 13:**
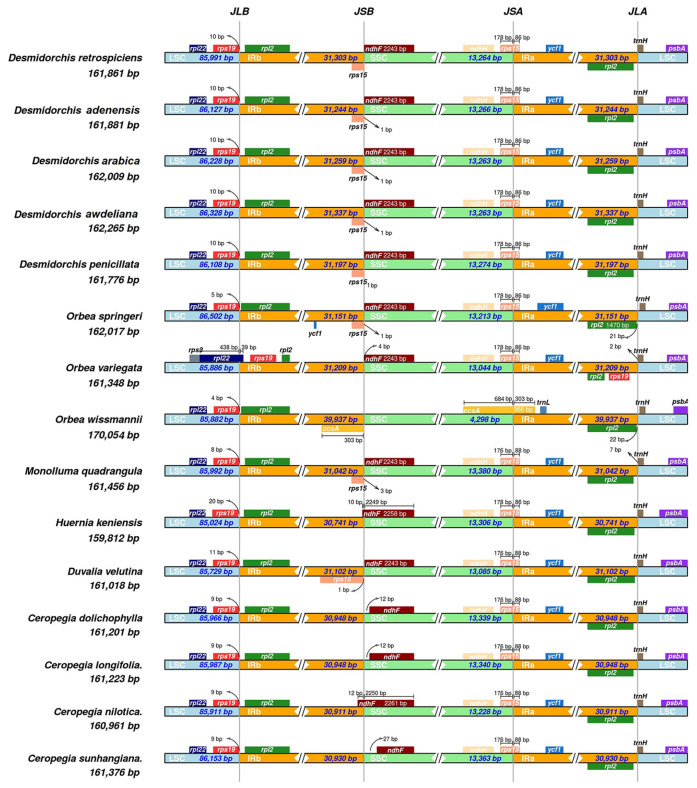
Structural comparison of the inverted repeat and single-copy (IR/SC) junctions in fifteen Stapeliinae chloroplast genomes. This diagram illustrates the positions of genes flanking the four junctions: JLB (LSC/IRb), JSB (IRb/SSC), JSA (SSC/IRa), and JLA (IRa/LSC).

The analysis of IR junctions across fifteen Stapeliinae chloroplast genomes revealed both conserved and variable patterns (Figure 13). *Desmidorchis* species exhibited a highly conserved plastome structure, with no structural variation observed among the five taxa. The IRa–LSC junction (JLA) was completely conserved across all Stapeliinae species, consistently flanked by *trnH*, reflecting strong structural stability at this boundary. In contrast, the LSC–IRb junction (JLB) showed minor variation. The *rps19* gene bordered IRb in all species except *Orbea variegata*, where a slight expansion of IRb into the LSC shifted the position of *rps19* inward.

The SSC–IRa junction (JSA) was generally characterized by the expansion of IRa into the SSC to include the entire *ycf1* gene. In most species, JSA also spanned 86–88 bp of the *rps15* gene, resulting in the formation of a partial duplicate (*rps15* pseudogene) in the IRb region. A notable exception was found in *O. wissmannii*, where the IRa expanded further to span 303 bp of the *ccsA* gene—an uncommon structural feature not observed in other taxa. The IRb–SSC junction (JSB) was relatively stable, typically bordered by *ndhF*. However, in *Ceropegia nilotica* and *Huernia keniensis*, a slight expansion of IRb into the SSC (10–12 bp) resulted in the inclusion of a portion of *ndhF* within the junction, thereby shifting its boundary position.

#### 3.2.6. Codon Usage Patterns

Relative synonymous codon usage (RSCU) analysis was performed on the chloroplast genomes of *Desmidorchis* and related taxa within the subtribe Stapeliinae (Table S10 and Figure S2). Stop codons (UAA, UAG, and UGA), which do not encode amino acids, were excluded from the analysis. Among the 61 sense codons, the most frequently used codon across the fifteen examined species was GAA, encoding glutamic acid (Glu). Its frequency ranged from 918 in *Orbea sprengeri* to 964 in *Monolluma quadrangula*, with a total count of 14,287 and RSCU values between 1.52 and 1.54. In contrast, the least frequently used codon was UGC, encoding cysteine (Cys), with counts ranging from 72 to 73, a total of 1091 occurrences, and RSCU values between 0.57 and 0.60.

Of the 61 sense codons, 29 exhibited RSCU values greater than 1, indicating preferential usage, with values ranging from 1.02 to 1.64. Notably, 28 of these preferred codons ended with A or U, while only one ended with G, highlighting a strong bias toward A/U-ending codons in Stapeliinae plastomes. Codons such as UUA (Leucine), AGA (Arginine), UCU (Serine), GCU (Alanine), and UAU (Tyrosine) showed particularly high RSCU values (>1.60), suggesting a strong codon preference. Conversely, 30 codons had RSCU values less than 1 (0.39–0.94), indicating underrepresentation. Among these, 28 codons ended with G or C, while only 2 (AUA and CUA) ended with A, further supporting the preference for A/U-ending codons and the relative avoidance of G/C-ending codons. Additionally, the codons AUG (methionine) and UGG (tryptophan) both exhibited RSCU values equal to 1, reflecting the absence of codon usage bias for these amino acids, each of which is encoded by a single codon.

**Figure 14:**
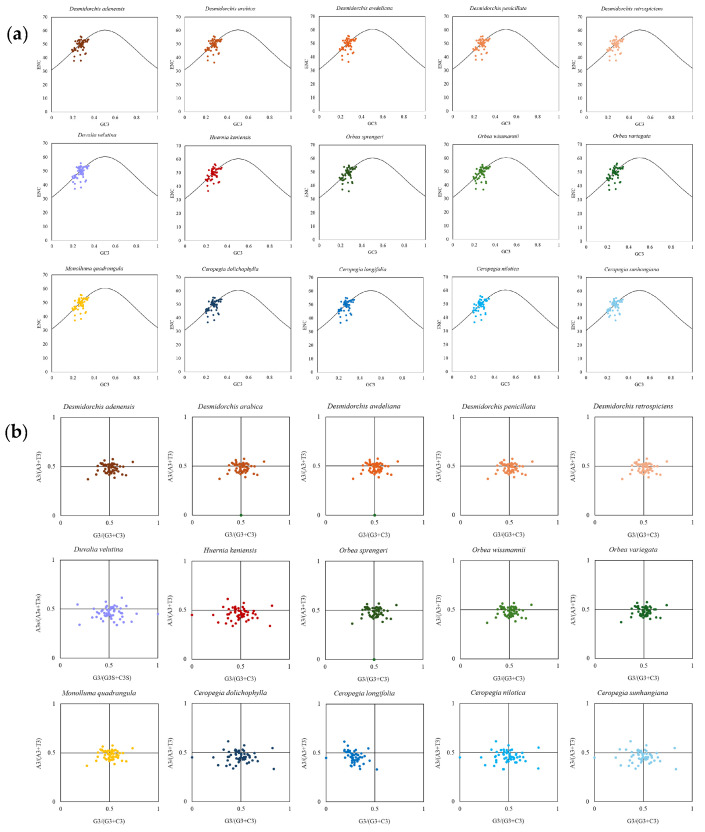
Codon usage bias analyses across the examined Stapeliinae plastomes. Different colors represent the analyzed taxa. (**a**) ENC–GC3 plots showing the relationship between GC3 content (*x*-axis) and effective number of codons (ENC) values (*y*-axis) for retained protein-coding genes in each species. (**b**) PR2-bias plots illustrating the distribution of fourfold degenerate codons, with G3/(G3 + C3) on the *x*-axis and A3/(A3 + T3) on the *y*-axis for each analyzed plastome.

The ENC–GC3 analysis (Figure 14a and Table S11) demonstrated that most protein-coding genes were distributed below the expected curve, with ENC values generally exceeding 35. This pattern indicates that codon usage bias in these plastomes is relatively weak but cannot be explained solely by mutational pressure. The deviation of genes from the expected curve suggests that natural selection, in addition to mutation, contributes to shaping codon usage patterns. PR2 plot analysis (Figure 14b and Table S12) further supported this observation. The distribution of genes around the central point (0.5, 0.5) was not perfectly symmetrical, indicating an imbalance between complementary nucleotides at the third codon position. Although the deviation from equilibrium was moderate, it suggests that both mutational bias and selective constraints jointly influence codon usage in these chloroplast genomes.

#### 3.2.7. Selective Pressure on Protein-Coding Genes

The Ka/Ks values of 79 unique chloroplast protein-coding genes were evaluated through pairwise comparisons across 15 Stapeliinae plastomes to assess selective pressure. After excluding genes with non-estimable values (NaN), 33 genes retained informative Ka/Ks estimates and were included in the final heatmap analysis (Figure S3). Most genes exhibited Ka/Ks ratios below 1 across the examined comparisons, indicating that purifying selection is the predominant evolutionary force acting on Stapeliinae plastid genes.

The heatmap revealed generally conserved evolutionary patterns among *Desmidorchis*, *Orbea*, *Ceropegia*, *Monolluma*, *Duvalia*, and *Huernia*. Most retained photosynthesis-related genes, particularly members of the *psb*, *ndh*, and ATP synthase gene families, displayed relatively low Ka/Ks ratios, consistent with strong functional conservation. Similarly, housekeeping genes such as *rbcL*, *rpoA*, and *rpoB* generally showed low substitution ratios, although moderate increases were occasionally observed in *rpoC1* and *rpoC2*.

Despite the predominance of purifying selection, several genes exhibited comparatively elevated Ka/Ks values in specific pairwise comparisons. Among these, *clpP1* showed the highest and most consistent elevation across numerous taxon pairs, whereas elevated but comparatively lower values were also observed in *accD*, and occasionally in *infA*, *matK*, and *rpl22*. These patterns suggest accelerated sequence evolution or relaxed selective constraints affecting a limited subset of plastid loci.

Within *Desmidorchis*, comparisons among *D. adenensis*, *D. arabica*, *D. awdeliana*, *D. penicillata*, and *D. retrospiciens* showed broadly similar Ka/Ks profiles dominated by low substitution ratios, reflecting strong plastome conservation within the genus. Several loci produced undefined values (gray cells), particularly among closely related *Desmidorchis* taxa, due to the absence of synonymous substitutions (Ks = 0). In contrast, comparisons between *Desmidorchis* species and other Stapeliinae genera frequently showed elevated Ka/Ks values in *clpP1* and *accD*, suggesting that these loci could contribute disproportionately to intergeneric evolutionary divergence within the subtribe. Overall, the results indicate that purifying selection predominates across Stapeliinae plastomes, whereas a limited number of genes, particularly *clpP1*, exhibit signatures of accelerated evolution or relaxed selective pressure.

### 3.3. Phylogenomic Relationships

**Figure 15:**
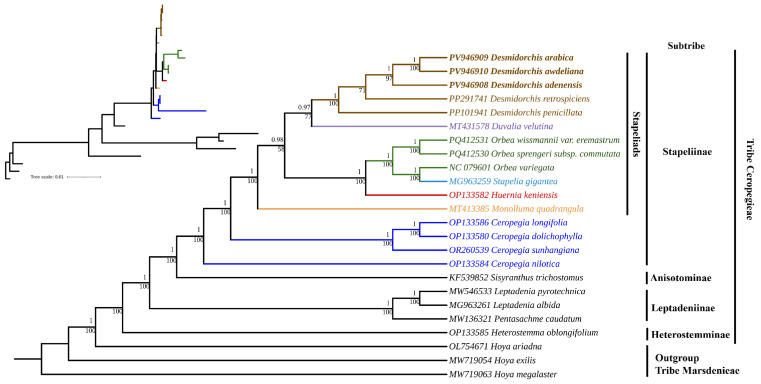
Maximum likelihood (ML) tree of 24 Ceropegieae species and three outgroups based on 80 protein-coding genes. ML bootstrap values are shown below branches, and Bayesian posterior probabilities (PP) are shown above. Subtribe Stapeliinae genera are color-coded as follows: *Desmidorchis* (brown), *Orbea* (green), *Huernia* (red), *Monolluma* (orange), *Duvalia* (violet), *Stapelia* (light blue), and *Ceropegia* (blue). The newly sequenced *Desmidorchis* species are highlighted in bold brown.

Both maximum likelihood (ML) and Bayesian inference (BI) analyses of the 80 CDS supermatrix yielded a fully resolved phylogeny of tribe Ceropegieae with near-universal maximal support (ML bootstrap values, BS, and Bayesian posterior probabilities, PP). The ML tree is presented in the main text (Figure 15), while the BI tree is provided in the Supplementary Materials (Figure S4). The two trees were topologically identical except for the relationships between *D. penicillata* and *D. retrospiciens* (Figure 15 and Figure S4). All five *Desmidorchis* plastomes formed a strongly supported monophyletic clade (BS/PP = 100/1.00). The three newly sequenced species grouped together: *D. awdeliana* and *D. arabica* were recovered as sisters (BS/PP = 100/1.00), with *D. adenensis* sister to that pair (BS/PP = 97/1.00). The two previously published species comprised the remaining lineage. In the ML tree (Figure 15), *D. retrospiciens* was recovered as sister to the trio of newly sequenced taxa with moderate bootstrap support (BS = 71), while *D. penicillata* occupied the basal position as sister to all other *Desmidorchis*. In contrast, the BI tree (Figure S4) did not resolve the relationships between *D. penicillata* and *D. retrospiciens*; instead, they formed a polytomy with the clade of the three Arabian species.

The *Desmidorchis* clade was placed as the immediate sister to *Duvalia velutina* (BS/PP = 77/0.97), together forming the first-diverging branch of the “core stapeliad” assemblage. The second branch comprised plastomes of *Huernia*, *Stapelia*, and *Orbea*, while *Monolluma quadrangula* formed a grade sister to all stapeliad taxa. The *Ceropegia* species (*C. longifolia*, *C. dolichophylla*, and *C. sunhangiana*) likewise formed a distinct and strongly supported clade that was consistently sister to the stapeliads. Meanwhile, *C. nilotica* diverged earlier and recovered as sister to the entire subtribe Stapeliinae (BS/PP = 100/1.00). Representatives of Leptadeniinae, Heterostemminae, and Anisotominae were placed in well-supported positions outside Stapeliinae.

### 3.4. Evolutionary Divergence Times

The molecular clock analysis, conducted at the subfamily Asclepiadoideae level using fossil calibration points, established a temporal framework for the evolutionary radiation of the studied taxa. The resulting chronogram (Figure S5) supports a late Eocene origin for the tribe Ceropegieae at approximately 41.81 million years ago (Ma) (95% HPD: 34.95–48.94 Ma), followed by tribal diversification during the late Eocene at 35.28 Ma (95% HPD: 27.92–42.34 Ma).

Within the Ceropegieae, the diversification of the stapeliad lineage initiated during the late Miocene, at approximately 6.8 Ma (95% HPD: 4.51–9.48 Ma). Subsequent radiation within the genus *Desmidorchis* occurred more recently, primarily during the Pleistocene at 1.63 Ma (95% HPD: 0.63–2.25 Ma). The divergence among the three focal endemics—*D. adenensis*, *D. arabica*, and *D. awdeliana*—similarly took place during the Pleistocene, between 0.34 and 1.51 Ma. Within this clade, *D. adenensis* emerged as the most basal lineage, diverging at approximately 0.8 Ma. The subsequent split between *D. arabica* and *D. awdeliana* was estimated at 0.2 Ma. 

## 4. Discussion

The southern Arabian Peninsula is a key center of succulent diversity, supporting numerous endemic taxa that contribute to its status as a biodiversity hotspot [1,2,89]. Within this context, species of the genus *Desmidorchis* constitute an important component of the regional flora, with eight of the approximately fourteen known species restricted to the Arabian Peninsula [4]. Many of these species have documented ethnomedicinal uses [7], which, together with increasing anthropogenic pressures [6,17,18], may contribute to population declines and local extinctions in parts of their range. In particular, this study focuses on three ethnomedicinally important species—*D. adenensis*, *D. arabica*, and *D. awdeliana*—which are widely used and potentially subject to increasing exploitation pressure.

Despite their ecological and cultural importance, the global conservation status of Arabian *Desmidorchis* species continues to be poorly resolved, as most extinction risk assessments have been conducted at regional rather than global scales [6,19,20]. In addition, Arabian endemic plants are still underrepresented in genomic studies, despite the growing number of plastome-based investigations in recent years [36]. As a result, the evolutionary history and temporal diversification of these lineages remain largely unexplored.

This study represents an important step toward addressing these knowledge gaps by integrating global-scale IUCN Red List assessments with plastome-based genomic analyses and molecular clock dating. This combined approach provides updated evidence-based insights into species distribution, conservation status, and evolutionary history, while improving our understanding of how past climatic events may have shaped present-day biodiversity patterns in the Arabian Peninsula.

### 4.1. Conservation Implications for Arabian Desmidorchis

The conservation assessment conducted here indicates that these species qualify as Near Threatened (NT). This category is assigned to species that do not yet meet the criteria for threatened categories (Critically Endangered, Endangered, or Vulnerable) but are close to qualifying or are likely to qualify in the near future [41]. It therefore serves as an early warning signal, indicating that ongoing monitoring and conservation measures are required to prevent further decline. The primary drivers underlying this classification include habitat loss, overexploitation, and climate change [6,17,18], all of which are widely recognized as major drivers of biodiversity loss in arid and semi-arid ecosystems [90,91]. Notably, Yemen represents a key center of distribution for the studied species (Figure 3), harboring all taxa included in this assessment. However, plant diversity in Yemen is increasingly threatened by a combination of natural and anthropogenic pressures, including habitat degradation and land-use change [92].

These pressures may accelerate population decline and potentially lead to the future reclassification of these species into higher threat categories. Consequently, effective conservation of Arabian *Desmidorchis* requires coordinated regional efforts among Arabian Peninsula countries, particularly those sharing transboundary populations, to ensure the long-term persistence of these ecologically and culturally significant species. The spatially explicit data generated in this study provide a robust foundation for guiding such conservation planning and management efforts.

Beyond conservation assessment, plastome-scale genomic data generated in this study offer new insights into the structural evolution, sequence variation, and divergence timing of *Desmidorchis* species. The following sections focus on the comparative analysis of chloroplast genome architecture, genomic features, and the evolutionary radiation of the genus within the Arabian Peninsula.

### 4.2. Chloroplast Genome Structure and Organization

The plastid is a photosynthetic organelle containing an autonomous genome, typically organized into a conserved quadripartite structure consisting of LSC, SSC, and two IR regions [30,31,32]. The plastomes of *Desmidorchis* exhibit the same quadripartite organization and a high degree of conservation. Their genomic architectures, GC contents, and gene orders closely resemble those reported for other Apocynaceae species and align with those of typical eudicot plastomes [32,37,40,93,94,95,96,97,98,99,100,101]. Total plastome sizes for *Desmidorchis* range from 161,881 bp to 162,265 bp, falling within the reported range for the family [40,102] and remaining comparable to the average plastome size in eudicots [103]. Each plastome encoded 114 unique genes, which is consistent with the typical gene content (110–130 genes) reported for most angiosperm chloroplast genomes involved in photosynthesis, transcription, and translation processes [30,31,32].

Analysis of gene structure revealed the presence of 18 unique intron-containing genes in each plastome, predominantly located within the LSC and IR regions. This pattern of intron distribution is consistent with that reported across diverse angiosperm lineages [31,32,104]. Several conserved structural features typical of land plant plastomes were also observed, including the presence of two introns in both *clpP1* and *ycf3*, as well as a large intron (~2524 bp) within the *trnK-UUU* gene [105,106,107,108]. Characterizing intron–exon structures provides critical insights into RNA splicing mechanisms and genome evolution, while offering valuable markers for phylogenetic reconstruction [109,110].

The genomes are predominantly A/T-rich, with this fraction comprising up to 62% of the sequence, a trend consistent with other Apocynaceae members [40]. Additionally, the GC content within the IR regions is significantly higher than that of the single-copy (LSC and SSC) regions. This phenomenon, widely documented in higher plants, is largely attributed to the presence of GC-rich ribosomal RNA (rRNA) and transfer RNA (tRNA) genes localized within the IR regions [32]. Overall, the high level of structural conservation observed among *Desmidorchis* plastomes reflects evolutionary stability within the genus and supports their close phylogenetic affinity within Apocynaceae, while providing a reliable genomic framework for comparative and evolutionary analyses.

### 4.3. Repeat Sequence Variation

Repetitive sequences are a hallmark of chloroplast genomes, driving the genetic variation necessary for evolutionary and population-level research [111,112,113]. Each repeat type arises through distinct mutational mechanisms and exhibits unique patterns of polymorphism [114,115,116]. In this study, Simple Sequence Repeats (SSRs), tandem repeats, and dispersed (long) repeats were analyzed across fifteen Stapeliinae plastomes to evaluate their distribution and potential informative value within Arabian *Desmidorchis* species.

As a powerful type of molecular marker, SSRs are widely utilized across botanical research due to their high polymorphism and cost-effectiveness [117,118,119]. Chloroplast SSRs have been extensively used in genetic diversity assessments, species identification, and phylogenetic studies due to their codominant inheritance, high information content, broad genomic distribution, and ease of detection [120,121]. Importantly, plastome-derived SSRs provide an economical and effective tool for tracing uniparental gene flow and inferring historical demographic events such as population bottlenecks and genetic drift [118].

The analysis of cpSSRs in this study confirms that SSR distribution is non-random; they primarily cluster within regions of lower selective pressure, such as intergenic spacers and introns within the LSC and SSC regions, in accordance with previous reports [61,122,123]. Mononucleotide SSRs were the most abundant repeat type in all plastomes analyzed, with A/T motifs predominating, a pattern widely reported in numerous plant lineages [101,106,124,125].

Despite these general similarities, specific characteristics of SSRs—including number, size, and motif profile—exhibited distinct patterns among the studied *Desmidorchis* taxa (Figure 5, Figure 6 and Figure 8). The genus *Desmidorchis* appears to maintain a high level of genomic stability, with a nearly uniform SSR abundance (0.46–0.48 No./kb) and a tightly clustered relative density (5.56–5.97 bp/kb) across the five analyzed species. This pattern is further characterized by a consistently low GC content (0.03–0.04). Based on our comparative analysis, several potentially informative sequence features were identified, including a hexanucleotide motif (AAATTA) within the *trnN-GUU*-*ycf1* intergenic spacer and an ATTA motif in the IGS-*psbM* region. Similar lineage-associated SSR patterns and motif distributions have previously been reported in plastome studies [61,126,127,128]. However, broader intraspecific and interspecific sampling is needed to confirm their discriminatory value across *Desmidorchis*.

Beyond SSRs, various repeat structures—including forward, palindromic, reverse, and tandem repeats—were identified in the examined plastomes. These regions are frequently characterized as hotspots for genome reconfiguration [129,130,131,132]. In agreement with previous reports [105,133,134], complementary repeats were consistently infrequent across all species. Forward and palindromic repeats were found to be the dominant long-repeat types, with their distribution patterns remaining highly similar across the species.

Tandem repeats, however, revealed variation within *Desmidorchis*. While the total repeat count remains stable across *D. adenensis* (106), *D. penicillata* (106), and *D. retrospeciens* (104), a notable expansion was observed in *D. awdeliana* (113) compared to *D. arabica* (97). This expansion is primarily concentrated within the LSC and IR regions (Table S7). Such interspecific variations are frequently attributed to slipped-strand mispairing and unequal crossover events [114,115,116]. Given the recent Pleistocene radiation of these lineages (~0.34–1.51 Ma), these repeat signatures may reflect structural variation associated with historical isolation in mountain refugia. Although based on limited sampling, the observed patterns suggest genomic divergence that warrants broader population-level investigation to determine whether these expansions represent stable lineage-specific markers.

### 4.4. Sequence Divergence and Highly Variable Regions

The chloroplast genome generally evolves slowly, but certain loci exhibit accelerated evolutionary rates, reflected by elevated nucleotide diversity (π) and frequent insertion–deletion events [104,135,136]. These hypervariable regions provide valuable sources of molecular markers and have been widely used in the development of DNA barcodes—standardized sequences for species identification [137,138]. Beyond taxonomic applications, plant DNA barcodes are increasingly used in forensic investigations, particularly for regulating the trade of endangered species and monitoring plant-derived commercial products such as foods and herbal supplements [139]. However, commonly used plastid barcoding loci, such as *matK* and *rbcL*, often lack sufficient resolution to discriminate among closely related species or hybrids [140,141]. Consequently, the concept of a “super-barcode,” based on the complete chloroplast genome or its most variable regions, has gained increasing attention in recent years [142,143,144].

In the present study, four loci—*ycf1*, *clpP1*, *ycf1-trnN*, and *psbM-trnD*—were identified as the most variable regions (π = 0.00582–0.033) across the analyzed Stapeliinae plastomes. These regions correspond to divergence hotspots previously reported in other plastome studies of Apocynaceae [37,40,95,101]. While conventional plastid markers (e.g., *matK*, *rbcL*, *trnH-psbA*, and *trnL-trnF*) have been widely evaluated for species discrimination in Apocynaceae [141,145,146,147,148,149,150,151], the loci identified here remain unexplored as potential DNA barcodes.

To assess their phylogenetic informativeness, the identified hypervariable regions were analyzed individually and in combination. Most loci showed only weak to moderate phylogenetic resolution when evaluated separately, reflecting the generally conserved nature of Stapeliinae plastomes. Among the tested datasets, the combined *ycf1* + *clpP1* region provided the strongest phylogenetic signal and produced a topology broadly congruent with the 80-CDS supermatrix tree. Previous studies have similarly recognized *ycf1* as one of the most informative plastid loci for phylogenetic reconstruction and species discrimination across angiosperms [152,153], providing both a strong phylogenetic signal and effective species-level discrimination [154,155,156]. Likewise, *clpP1* is known to evolve relatively rapidly in several angiosperm lineages [135] and has been explored as a phylogenetically informative marker in groups such as *Prunus* [157], *Dracaena* [158] and members of Actinidiaceae [159].

Despite the improved performance of the combined *ycf1* + *clpP1* dataset, BLASTn analyses indicated that these regions remain highly conserved within *Desmidorchis* and still exhibit high sequence similarity with closely related Stapeliinae genera. This high degree of similarity likely reflects the overall recent divergence and conserved plastome evolution within the subtribe. Consequently, although these loci retain a useful phylogenetic signal for resolving broader relationships within Stapeliinae, they may have limited utility as stand-alone diagnostic markers for direct genus-level identification of *Desmidorchis*. These findings highlight the need for future studies incorporating broader taxon sampling, SNP-based approaches, and finer-scale comparative analyses to identify shorter and more discriminatory molecular regions suitable for robust lineage-specific marker development and conservation applications.

### 4.5. Expansion and Contraction of Inverted Repeat (IR) Regions

The junctions between the IR regions and the LSC and SSC regions represent some of the most dynamic structural features of the chloroplast genome. These boundary regions frequently undergo expansion or contraction events [33,160]. Such IR boundary shifts are a major driver of plastome size variation among closely related taxa and can result in gene duplication, pseudogene formation, or relocation of genes between genomic regions [40,106,161,162]. Consequently, detailed analyses of IR boundary dynamics provide critical insights into plastome structural evolution and phylogenetic divergence.

In the present study, *Desmidorchis* species exhibited a highly conserved plastome structure, with no detectable variation in IR boundary organization among the five analyzed taxa. However, compared to the typical angiosperm plastome—where usually only a small portion (approximately 1000 bp) of the *ycf1* gene is duplicated within the IR [163]—*Desmidorchis* shows a significant IR expansion. Specifically, the IRa region expands into the SSC to encompass the entire *ycf1* gene and a portion of the *rps15* gene. This expansion results in the formation of a truncated duplicated fragment (*rps15* pseudogene) in the IRb region. This configuration is consistent with patterns reported in other Stapeliinae plastomes, including *Ceropegia longifolia*, *C. nilotica*, *C. dolichophylla*, *Huernia keniensis*, *Orbea variegata* [40], *C. sunhangiana* [164], *O. sprengeri* subsp. *commutata* [101], and *Monolluma quadrangula* [95]. Furthermore, the IR/LSC boundaries were more conserved than the IR/SSC boundaries, a pattern also observed in other members of Apocynaceae [37,40,95]. Ultimately, the stable IR boundary architecture observed across the studied *Desmidorchis* taxa reflects a high degree of structural stability and supports the evolutionary coherence of this lineage within the Stapeliinae.

### 4.6. Codon Usage Patterns and Selective Constraints

The codon usage analyses revealed broadly conserved patterns across the examined Stapeliinae plastomes, indicating a high degree of evolutionary conservation at the coding-sequence level. Preferred synonymous codons were predominantly A/U-ending, reflecting the AT-rich nature of plastid genomes, particularly at the third codon position. Similar A/U-biased codon usage patterns have been widely reported in angiosperm chloroplast genomes [99,105,106,165] and are generally associated with nucleotide compositional bias and plastome evolutionary constraints. The relatively similar RSCU profiles, ENC distributions, and PR2-plot patterns observed among the analyzed taxa further support the conserved nature of codon usage across Stapeliinae plastomes.

Codon usage bias is shaped by multiple evolutionary and molecular factors, including mutational tendencies, natural selection, translational efficiency, and nucleotide composition [166,167,168]. In the present study, ENC-plot and PR2-bias analyses suggested that mutational pressure alone could not fully explain the observed codon usage patterns. Most genes deviated from the expected ENC curve and showed unequal usage of complementary bases at the third codon position, indicating that natural selection, together with compositional constraints, contributes substantially to codon preference in Stapeliinae plastomes. These findings are consistent with previous reports from diverse angiosperm plastomes [169,170], where purifying selection has been recognized as a major factor shaping plastid coding-sequence evolution.

The Ka/Ks analyses further supported the highly conserved nature of Stapeliinae plastomes. Most retained protein-coding genes exhibited Ka/Ks ratios below 1 across pairwise comparisons, indicating the predominance of purifying selection and functional conservation among the examined taxa. Photosynthesis-related genes, particularly members of the *psb*, *ndh*, and ATP synthase gene families, generally displayed low substitution ratios, reflecting strong selective constraints associated with essential plastid functions. Similarly, housekeeping genes such as *rbcL*, *rpoA*, and *rpoB* remained highly conserved across most comparisons.

Despite the overall conservation of plastid genes, a limited number of loci exhibited comparatively elevated Ka/Ks values. Among these, *clpP1* showed the strongest and most consistent elevation across multiple intergeneric comparisons, while *accD* also displayed relatively increased substitution ratios in several taxon pairs. Both genes are known to evolve more rapidly than most plastid genes in many angiosperm lineages and have frequently been associated with relaxed selective constraints, structural variation, or lineage-specific adaptive processes [136,171]. In contrast, pairwise comparisons among closely related *Desmidorchis* species frequently yielded undefined values due to the absence of synonymous substitutions, further emphasizing the high sequence similarity and recent divergence within the genus.

Collectively, the concordance between codon usage conservation and the predominance of purifying selection highlights the evolutionary stability of Stapeliinae plastomes. Nevertheless, the comparatively elevated evolutionary rates observed in *clpP1* and *accD* suggest that these genes may contribute disproportionately to plastome divergence and lineage differentiation within the subtribe.

### 4.7. Phylogenomic Relationships and Temporal Diversification

Genome-wide patterns of variation provide powerful insights into evolutionary relationships and can reveal the phylogenetic structure with high precision [172]. In plants, the chloroplast genome is particularly valuable for phylogenetic reconstruction due to its structural stability and uniparental inheritance, which facilitates reliable sequence alignment while retaining sufficient variation to resolve relationships among closely related species [173,174,175].

The plastome-based phylogeny conducted in this study recovered relationships within tribe Ceropegieae that are largely consistent with previous nuclear and plastid datasets [176,177]. Within this framework, *Desmidorchis* forms a distinct lineage nested within subtribe Stapeliinae, in agreement with earlier phylogenetic analyses [178,179,180]. However, the use of a plastome-scale dataset provides an improved resolution of relationships among species within the *Desmidorchis* clade. This placement is also consistent with traditional morphology-based classifications [181,182].

Integrating a temporal framework provides a preliminary perspective on the genus’s history that complements existing biogeographic hypotheses. The estimated origin of the Ceropegieae (~41.81 Ma) suggests a late Eocene diversification that predates some previous estimates based on multi-gene datasets [183,184]. Nevertheless, the subsequent radiation of the stapeliad lineage during the late Miocene (~6.8 Ma; 95% HPD: 4.51–9.48 Ma) is broadly consistent with previous studies [179,184,185], which proposed that most diversification within the group occurred during the last 8–10 million years. This relatively recent radiation could be linked to the evolution of succulent growth forms in response to increasing aridity across the Old World drylands [179].

The estimated diversification of the sampled *Desmidorchis* species during the Pleistocene (~0.34–1.51 Ma) is likewise consistent with the recent diversification patterns proposed for southern Arabian stapeliads [179]. These divergence times coincide with periods of intensified aridification across the Old World [186,187,188], which may have promoted habitat fragmentation and ecological isolation of succulent lineages within the Arabian Peninsula. Furthermore, these Pleistocene divergence estimates align with the biogeographic patterns described by Miller and Nyberg [1] and Gazanfar [2], who identified the mountains of the southern Arabia as important centers of endemism and potential refugial systems for xerophytic flora. During the climatic oscillations of the Quaternary, these montane habitats may have provided relatively stable environmental conditions that facilitated the persistence and isolation of succulent populations [1,2]. Such refugial dynamics may help explain the high levels of endemism observed among Arabian stapeliads. 

Interestingly, although the studied *Desmidorchis* species exhibit substantial variation in floral morphology, the molecular data indicates relatively shallow divergence times (~0.2–0.8 Ma). A similar pattern was noted by Bruyns et al. [180], who reported marked floral variation among *Desmidorchis* despite relatively limited variation in the investigated gene regions. In addition, Bruyns et al. [179] emphasized the remarkable floral plasticity within stapeliads compared with the greater coherence of vegetative characters. Together, these observations suggest that floral diversification in *Desmidorchis* may occur rapidly under localized ecological conditions and could potentially reflect differences in pollination syndromes or pollinator-related selective pressures. However, broader taxonomic sampling and nuclear genomic data will be necessary to clarify the evolutionary mechanisms underlying diversification within the genus.

## 5. Conclusions

This study presents the first integrated assessment of the conservation status, comparative plastome genomics, and phylogenomic relationships of Arabian *Desmidorchis*, focusing on three endemic species: *D. adenensis*, *D. arabica*, and *D. awdeliana*. The Near Threatened (NT) status assigned to the studied species highlights the growing vulnerability of these geographically restricted succulents to habitat disturbance and environmental change within the Arabian mountain systems.

Despite the overall structural stability of the plastomes, several coding and intergenic regions displayed elevated variability and phylogenetic signal, indicating their potential value for future taxonomic and evolutionary studies. Repeat sequence patterns, including SSRs, also revealed potentially informative genomic signatures within the genus. Analyses of codon usage and selective pressure demonstrated that plastid genome evolution in Stapeliinae remains largely constrained by purifying selection, with only a small number of loci contributing substantially to sequence divergence. In parallel, phylogenomic reconstruction and molecular dating indicate that the diversification of Arabian Desmidorchis is relatively recent and closely linked to Pleistocene climatic dynamics, supporting the role of Arabian mountain refugia in shaping present-day succulent diversity.

Collectively, these findings establish an important baseline for future genomic and conservation research on Arabian endemic plants. Nevertheless, the current plastome-based framework represents only part of the evolutionary history of the genus *Desmidorchis*. Integrating nuclear genomic datasets, broader geographic and population-level sampling, and SNP-based analyses will be necessary to better resolve recent diversification, introgression, hybridization, and lineage boundaries within *Desmidorchis*.

## Figures and Tables

**Table 1:** Comparison of chloroplast genomic features among *Desmidorchis* species.

Features	Species (Accession Number)				
	*D. adenensis* (PV946908)	*D. arabica* (PV946909)	*D. awdeliana* (PV946910)	*D. penicillata* (PP101941)	*D. retrospiciens* (PP291741)
Genome size (bp)	161,881	162,009	162,265	161,776	161,861
LSC length (bp)	86,125	86,226	86,326	86,107	85,990
SSC length (bp)	13,266	13,263	13,263	13,273	13,264
IR length (bp)	31,245	31,260	31,338	31,198	31,303
Coding sequences (bp)	85,458	85,410	85,353	85,494	85,611
Percentage of coding sequences (%)	52.7	52.7	52.6	52.8	52.7
Non-coding sequences (bp)	76,423	76,599	76,912	76,282	76,666
Percentage of non-coding sequences (%)	47.2	47.2	47.3	47.1	47.2
Total number of genes	133	133	133	133	133
Number of unique genes	114	114	114	114	114
Number of duplicated genes	19	19	19	19	19
Total number of protein-coding genes	88	88	88	88	88
Total number of tRNA genes	37	37	37	37	37
Total number of rRNA genes	8	8	8	8	8
Total AT content (%)	62.3	62.2	62.3	62.2	62.3
Total GC content (%)	37.7	37.8	37.7	37.8	37.7
GC content in LSC (%)	36.2	36.2	36.2	36.1	36.2
GC content in SSC (%)	32.8	32.8	32.8	32.8	32.8
GC content in IR (%)	41.0	41.0	40.9	41.0	41.0

**Table 2:** Gene composition of the chloroplast genomes of *Desmidorchis adenensis*, *D. arabica*, and *D. awdeliana*, including counts of protein-coding, tRNA, and rRNA genes.

Category	Group of Genes	Name of Genes	Number
Self-replication	Ribosomal RNA genes (rRNA)	*rrn5*^(*X2*)^, *rrn4.5*^(*X2*)^, *rrn16*^(*X2*)^, *rrn23*^(*X2*)^	8 (4)
	Transfer RNA genes (tRNA)	*trnA-UGC*^+,(*X2*)^, *trnC-GCA*, *trnD-GUC*, *trnE-UUC*, *trnF-GAA*, *trnfM-CAU*, *trnG-GCC*, *trnG-UCC*, *trnH-GUG*, *trnI-CAU*^(*X2*)^, *trnI-GAU*^+,(*X2*)^, *trnK-UUU*^+^, *trnL-CAA*^(*X2*)^, *trnL-UAA*^+^, *trnL-UAG*, *trnM-CAU*, *trnN-GUU*^(*X2*)^, *trnP-UGG*, *trnQ-UUG*, *trnR-ACG*^(*X2*)^, *trnR-UCU*, *trnS-GCU*, *trnS-GGA*, *trnS-UGA*, *trnT-GGU*, *trnT-UGU*, *trnV-GAC*^(*X2*)^, *trnV-UAC*^+^, *trnW-CCA*, *trnY-GUA*	37 (7)
	Small subunit of ribosome	*rps2*, *rps3*, *rps4*, *rps7*^(*X2*)^, *rps8*, *rps11*, *rps12*^+,(*X2*)^, *rps14*, *rps15*, *rps16*^+^, *rps18*, *rps19*	14 (2)
	Large subunit of ribosome	*rpl2*^+,(*X2*)^, *rpl14*, *rpl16*^+^, *rpl20*, *rpl22*, *rpl23*^(*X2*)^, *rpl32*, *rpl33*, *rpl36*	11 (2)
	DNA-dependent RNA polymerase	*rpoA*, *rpoB*, *rpoC1*^+^, *rpoC2*	4
Genes for photosynthesis	Photosystem I	*psaA*, *psaB*, *psaC*, *psaI*, *psaJ*, *ycf3*^++^, *ycf4*	7
	Photosystem II	*psbA*, *psbB*, *psbC*, *psbD*, *psbE*, *psbF*, *psbH*, *psbI*, *psbJ*, *psbK*, *psbL*, *psbM*, *psbN*, *psbT*, *psbZ*	15
	Subunits of cytochrome b/f complex	*petA*, *petB*^+^, *petD*^+^, *petG*, *petL*, *petN*	6
	Subunits of ATP synthase	*atpA*, *atpB*, *atpE*, *atpF*^+^, *atpH*, *atpI*	6
	Large subunit of rubisco	*rbcL*	1
	Subunits of NADH-dehydrogenase	*ndhA*^+^, *ndhB*^+,(*X2*)^, *ndhC*, *ndhD*, *ndhE*, *ndhF*, *ndhG*, *ndhH*, *ndhI*, *ndhJ*, *ndhK*	12 (1)
Other genes	Chloroplast envelope membrane protein	*cemA*	1
	Maturase	*matK*	1
	ATP-dependent protease subunit P	*clpP* ^++^	1
	Subunit acetyl-coA carboxylase	*accD*	1
	C-type cytochrome synthesis	*ccsA*	1
	Translational initiation factor	*infA*	1
	Hypothetical proteins	*ycf2*^(*X2*)^, *ycf15*^(*X2*)^	4 (2)
	Component of TIC complex	*ycf1* ^(*X2*)^	2 (1)
Total			133 (19)

^+^ Gene with one intron, ^++^ gene with two introns, ^(*X2*)^ indicates that the number of the repeat unit is 2, parentheses indicate the number of duplicated genes.

**Table 3:** Exon and intron lengths across *Desmidorchis* species (*D. adenensis*, *D. arabica*, and *D. awdeliana*).

Gene	Location	Exon 1	Intron 1	Exon 2	Intron 2	Exon 3
*atpF*	LSC	145	696	407		
*clpP1*	LSC	71	749	292	673	228
*ndhA*	SSC	553	1097	545		
*ndhB*	IR	777	684	756		
*petB*	LSC	6	776	642		
*petD*	LSC	8	850	475		
*rpl16*	LSC	9	928	405		
*rpl2*	IR	391	646	434		
*rpoC1*	LSC	432	771	1605		
*rps12*	LSC	114	534	232		26
*rps16*	LSC	40	835	227		
*trnA*-UGC	IR	38	817	35		
*trnG*-UCC	LSC	23	666	48		
*trnI*-GAU	IR	42	943	35		
*trnK*-UUU	LSC	37	2524	35		
*trnL*-UAA	LSC	37	481	50		
*trnV*-UAC	LSC	38	597	37		
*ycf3*	LSC	124	732	230	773	153

**Table 4:** Overview of SSR number, relative abundance, density, and GC content in fifteen chloroplast genomes of Stapeliinae.

Species	SSRs Characteristics					
	Sequence Analyzed (kb)	Number of SSRs	Relative Abundance (No./kb)	Total Length of SSRs (bp)	Relative Density (bp/kb)	GC Content
*Desmidorchis adenensis*	161.881	76	0.47	956	5.91	0.0334
*Desmidorchis arabica*	162.009	75	0.46	900	5.56	0.0356
*Desmidorchis awdeliana*	162.265	76	0.47	920	5.67	0.0348
*Desmidorchis penicillata*	161.776	78	0.48	966	5.97	0.0331
*Desmidorchis retrospiciens*	161,861	77	0.47	939	5.79	0.0447
*Orbea wissmannii*	171.105	83	0.49	1434	8.38	0.0377
*Orbea sprengeri*	162.017	66	0.41	841	5.19	0.063
*Orbea variegata*	161.348	62	0.38	771	4.78	0.067
*Huernia keniensis*	159.812	70	0.44	859	5.38	0.0664
*Duvalia velutina*	161.018	76	0.47	935	5.81	0.0439
*Monolluma quadrangula*	161.456	71	0.44	854	5.29	0.0644
*Ceropegia dolichophylla*	161.201	77	0.48	934	5.79	0.0685
*Ceropegia nilotica*	160.961	82	0.51	1009	6.27	0.0773
*Ceropegia longifolia*	161.223	78	0.48	942	5.84	0.0679
*Ceropegia sunhangiana*	161.376	82	0.51	990	6.13	0.0778

**Table 5:** The number, relative abundance, relative density, and GC content of mono- to hexanucleotide SSRs in fifteen Stapeliinae plastomes.

Repeat Type	Characteristics of SSR	Species														
		*D. adenensis*	*D. arabica*	*D. awdeliana*	*D. penicillata*	*D. retrospiciens*	*O. wissmannii*	*O. sprengeri*	*O. variegata*	*H. keniensis*	*Du. velutina*	*M. quadrangula*	*C. dolichophylla*	*C. nilotica*	*C. longifolia*	*C. sunhangiana*
Mono	No. of SSRs	52.0	51.0	52.0	53.0	52.0	47.0	40.0	43.0	50.0	54.0	45.0	49.0	55.0	51.0	53.0
	Abundance (No./kb)	0.32	0.31	0.32	0.33	0.32	0.27	0.25	0.27	0.31	0.34	0.28	0.30	0.34	0.32	0.33
	Density (bp/kb)	3.95	3.74	3.80	4.01	3.92	3.25	3.02	3.21	3.81	4.06	3.30	3.57	4.09	3.72	3.85
	GC content	0.02	0.02	0.02	0.02	0.03	0.02	0.02	0.04	0.04	0.02	0.04	0.04	0.05	0.04	0.05
Di	No. of SSRs	7.00	7.00	7.00	8.00	7.00	9.00	9.00	2.00	4.00	5.00	7.00	8.00	9.00	8.00	8.00
	Abundance (No./kb)	0.04	0.04	0.04	0.05	0.04	0.05	0.06	0.01	0.03	0.03	0.04	0.05	0.06	0.05	0.05
	Density (bp/kb)	0.44	0.44	0.44	0.52	0.44	0.58	0.62	0.12	0.26	0.34	0.46	0.55	0.70	0.53	0.55
	GC content	0.00	0.00	0.00	0.00	0.00	0.00	0.00	0.00	0.00	0.00	0.00	0.06	0.05	0.06	0.06
Tri	No. of SSRs	8.00	8.00	9.00	8.00	9.00	14.0	4.00	7.00	7.00	9.00	9.00	8.00	6.00	8.00	8.00
	Abundance (No./kb)	0.05	0.05	0.06	0.05	0.06	0.08	0.02	0.04	0.04	0.06	0.06	0.05	0.04	0.05	0.05
	Density (bp/kb)	0.68	0.61	0.68	0.61	0.59	2.74	0.30	0.59	0.53	0.75	0.67	0.60	0.45	0.60	0.59
	GC content	0.08	0.08	0.08	0.08	0.08	0.00	0.03	0.04	0.10	0.07	0.02	0.08	0.06	0.08	0.08
Tetra	No. of SSRs	6.00	6.00	6.00	6.00	6.00	7.00	9.00	8.00	7.00	6.00	7.00	7.00	8.00	7.00	9.00
	Abundance (No./kb)	0.04	0.04	0.04	0.04	0.04	0.04	0.06	0.05	0.04	0.04	0.04	0.04	0.05	0.04	0.06
	Density (bp/kb)	0.44	0.44	0.44	0.45	0.52	0.51	0.69	0.64	0.55	0.45	0.55	0.55	0.62	0.55	0.69
	GC content	0.17	0.16	0.16	0.16	0.14	0.04	0.03	0.13	0.15	0.13	0.04	0.15	0.19	0.15	0.14
Penta	No. of SSRs	1.00	1.00	0.00	1.00	1.00	2.00	1.00	1.00	0.00	0.00	1.00	1.00	2.00	0.00	0.00
	Abundance (No./kb)	0.01	0.01	0.00	0.01	0.01	0.01	0.01	0.01	0.00	0.00	0.01	0.01	0.01	0.00	0.00
	Density (bp/kb)	0.09	0.09	0.00	0.09	0.09	0.18	0.12	0.09	0.00	0.00	0.09	0.09	0.19	0.00	0.00
	GC content	0.00	0.00	0.00	0.00	0.00	0.00	0.00	0.00	0.00	0.00	0.08	0.00	0.20	0.00	0.00
hexa	No. of SSRs	2.00	2.00	2.00	2.00	2.00	4.00	3.00	1.00	2.00	2.00	2.00	4.00	2.00	4.00	4.00
	Abundance (No./kb)	0.01	0.01	0.01	0.01	0.01	0.02	0.02	0.01	0.01	0.01	0.01	0.02	0.01	0.02	0.02
	Density (bp/kb)	0.30	0.22	0.30	0.30	0.22	1.12	0.44	0.11	0.23	0.22	0.22	0.45	0.22	0.45	0.45
	GC content	0.00	0.00	0.00	0.00	0.00	0.02	0.06	0.50	0.33	0.33	0.03	0.21	0.33	0.21	0.21

## Data Availability

The data generated in this study are available in the article and Supplementary Materials. The whole chloroplast genome sequence of *D. adenensis*, *D. arabica* and *D. awdeliana* is available for download from GenBank at https://www.ncbi.nlm.nih.gov/ (accession NO.: PV946908, PV946909, PV946910, respectively). The raw Illumina sequencing reads have been deposited in the NCBI Sequence Read Archive (SRA) under BioProject ID: PRJNA1464182, with BioSample accession numbers SAMN59686055 (D. adenensis), SAMN59735311 (D. arabica), and SAMN59686056 (D. awdeliana).

## Supplementary Materials

The following supporting information can be downloaded at https://www.mdpi.com/article/10.3390/biology15100798/s1, Figure S1: Maximum likelihood (ML) tree of 24 Ceropegieae species based on hypervariable regions and their combinations; Figure S2: Heatmap analysis of codon distribution across all protein-coding genes for the species studied; Figure S3: Heatmap of pairwise Ka/Ks ratios for 33 chloroplast protein-coding genes across 15 Stapeliinae plastomes; Figure S4: Bayesian inference (BI) tree of 24 Ceropegieae species and three outgroups based on 80 protein-coding genes; Figure S5: Maximum clade credibility chronogram of Asclepiadoideae based on 62 shared chloroplast protein-coding genes; Table S1: Sequencing data metrics; Table S2: Chloroplast sequences used for molecular dating analysis; Table S3: List of simple sequence repeats; Table S4: The number, relative abundance and relative density of SSR motifs in the chloroplast genomes of fifteen Stapeliinae plastomes; Table S5: Frequency of classified repeat types (considering sequence complementary) in the chloroplast genomes of fifteen Stapeliinae; Table S6: Dispersed repeat sequences present in the chloroplast genomes of fifteen Stapeliinae; Table S7: The number and location of tandem repeat sequences in the chloroplast genomes of fifteen Stapeliinae; Table S8: The size of tandem repeat sequences in the chloroplast genomes of fifteen Stapeliinae; Table S9: Summary of BLASTn evaluation for the combined *clpP1* + *ycf1* dataset in three *Desmidorchis* species; Table S10: Putative preferred codons in the Stapeliinae chloroplast genomes; Table S11: The effective number of codons in the analysis of the Stapeliinae chloroplast genomes; Table S12: The parity rule 2 plot analysis of the Stapeliinae chloroplast genomes.

## Abbreviations

The following abbreviations are used in this manuscript:

AOO	Area of Occupancy
BS	Bootstrap Support
BI	Bayesian Inference
cpSSR	Chloroplast Simple Sequence Repeat
EOO	Extent of Occurrence
GC	Guanine–Cytosine Content
IGS	Intergenic Spacer
IR	Inverted Repeat
IRa/IRb	Inverted Repeat Regions a and b
IUCN	International Union for Conservation of Nature
LSC	Large Single-Copy Region
ML	Maximum Likelihood
NT	Near Threatened
CDS	Protein-Coding Sequences
Pi (π)	Nucleotide Diversity
PP	Posterior Probability
rRNA	Ribosomal RNA
SSC	Small Single-Copy Region
tRNA	Transfer RNA
