# Supplementary material for: Conservation Status, Plastome Diversity, and Evolutionary Diversification of Three Arabian Desmidorchis Endemics (Apocynaceae)
Source: Biology (Basel). 2026 May 17;15(10):798. doi: 10.3390/biology15100798 (PMC13203109; doi:10.3390/biology15100798)
Supplement: Supplementary file 1 [file biology-15-00798-s001.zip › Supplementary Materials Figurs S1-S5.pdf]

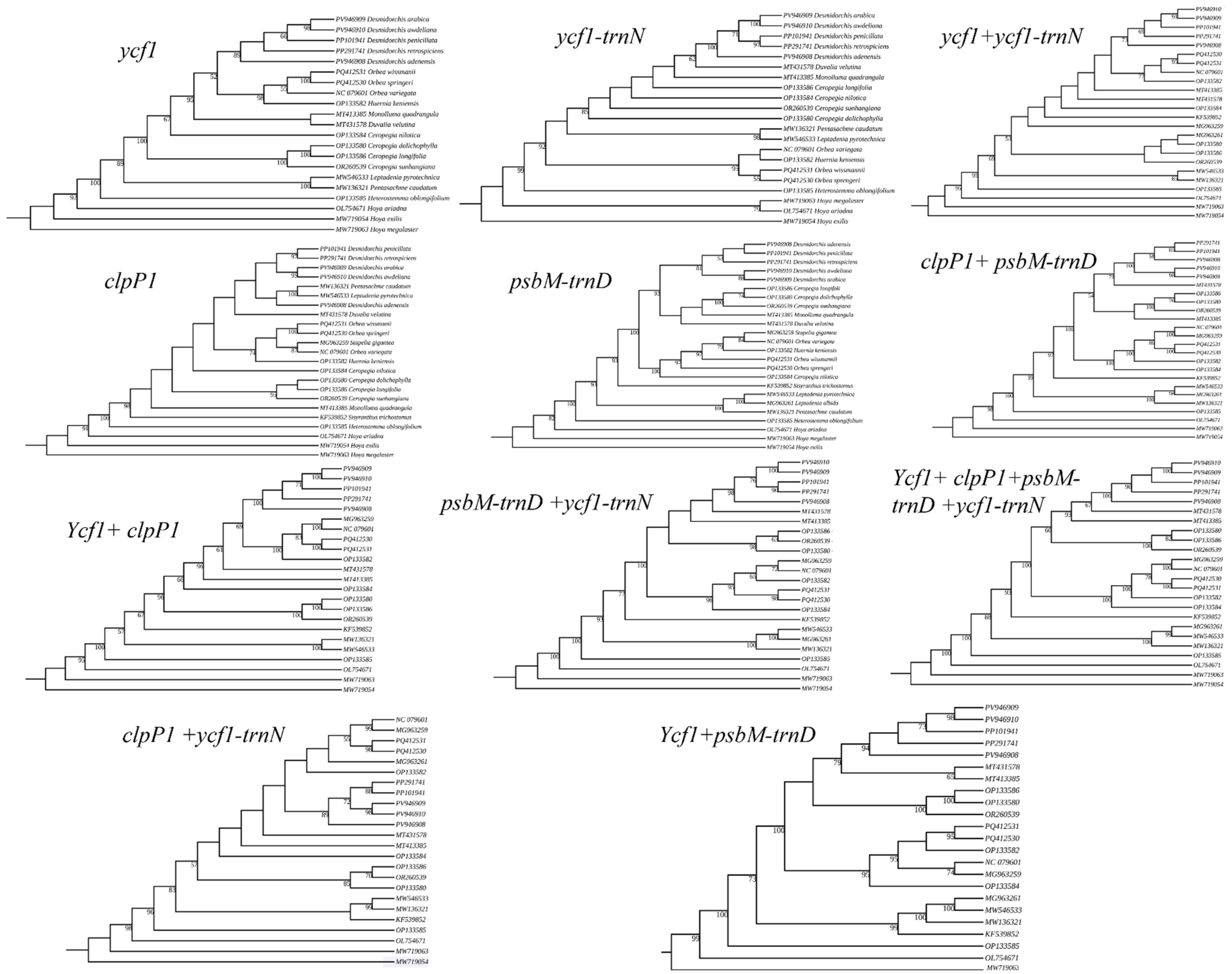

Figure S1 Maximum Likelihood (ML) tree of 24 Ceropegieae species based on hypervariable regions and their combinations

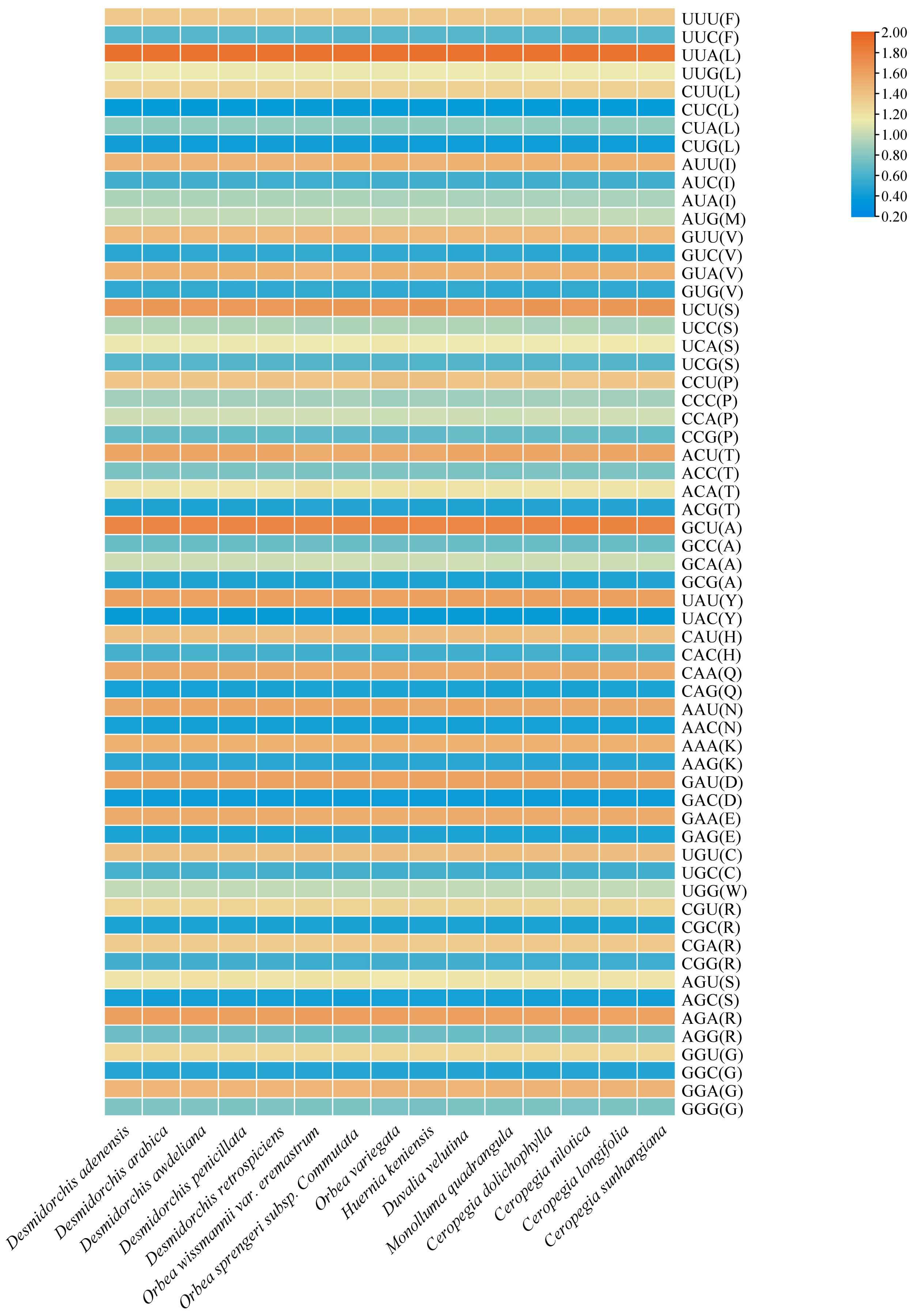

Figure S2. Heatmap analysis of codon distribution across all protein-coding genes for the species studied. Color key: Orange indicates higher RSCU values, while blue represents lower RSCU values.

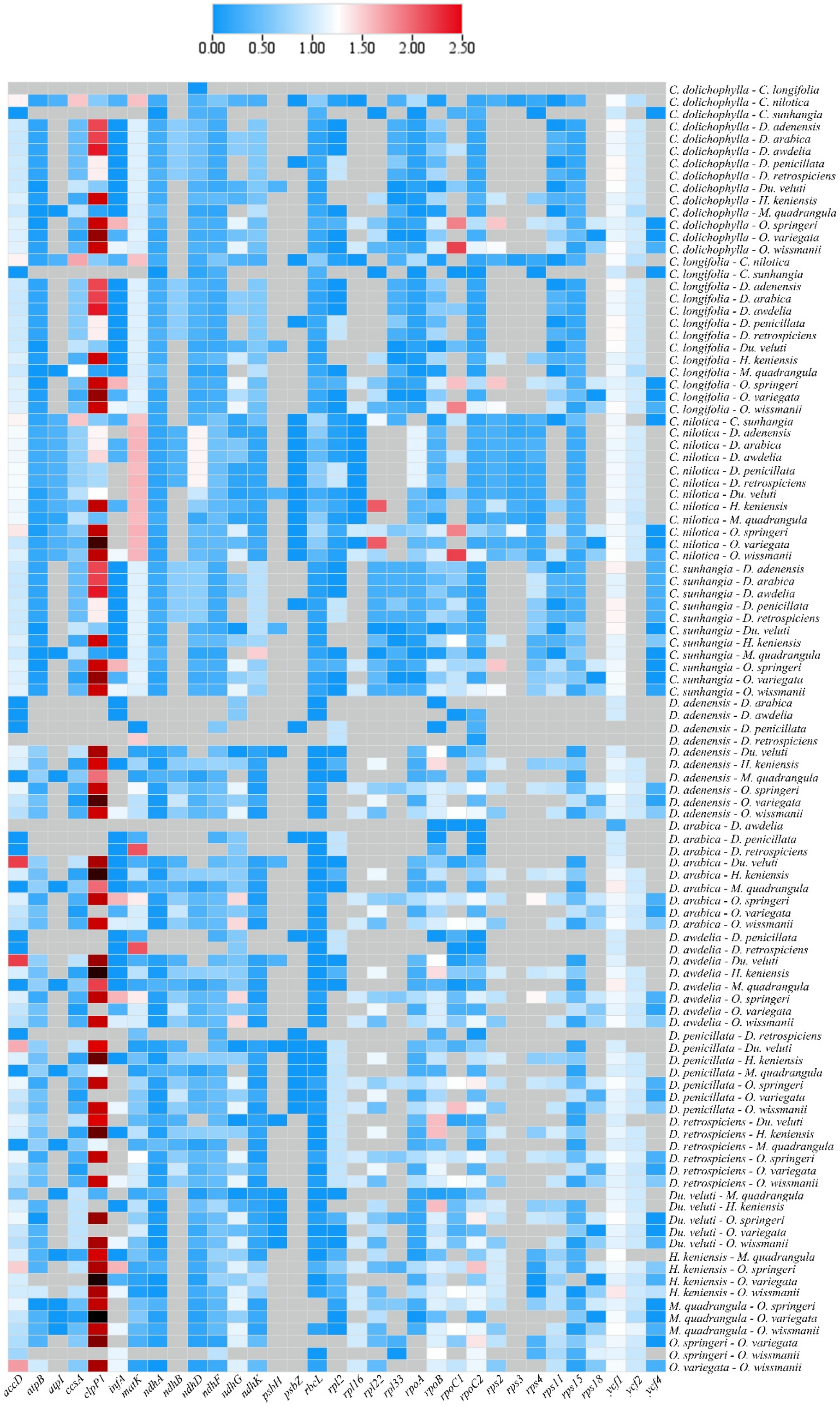

Figure S3. Heatmap of pairwise Ka/Ks ratios for 33 chloroplast protein-coding genes across 15 Stapeliinae plastomes. Gray cells represent undefined or non-estimable values (NaN or −Infinity) resulting from zero synonymous substitutions (Ks = 0). Black-colored cells indicate the highest observed Ka/Ks values in the dataset.

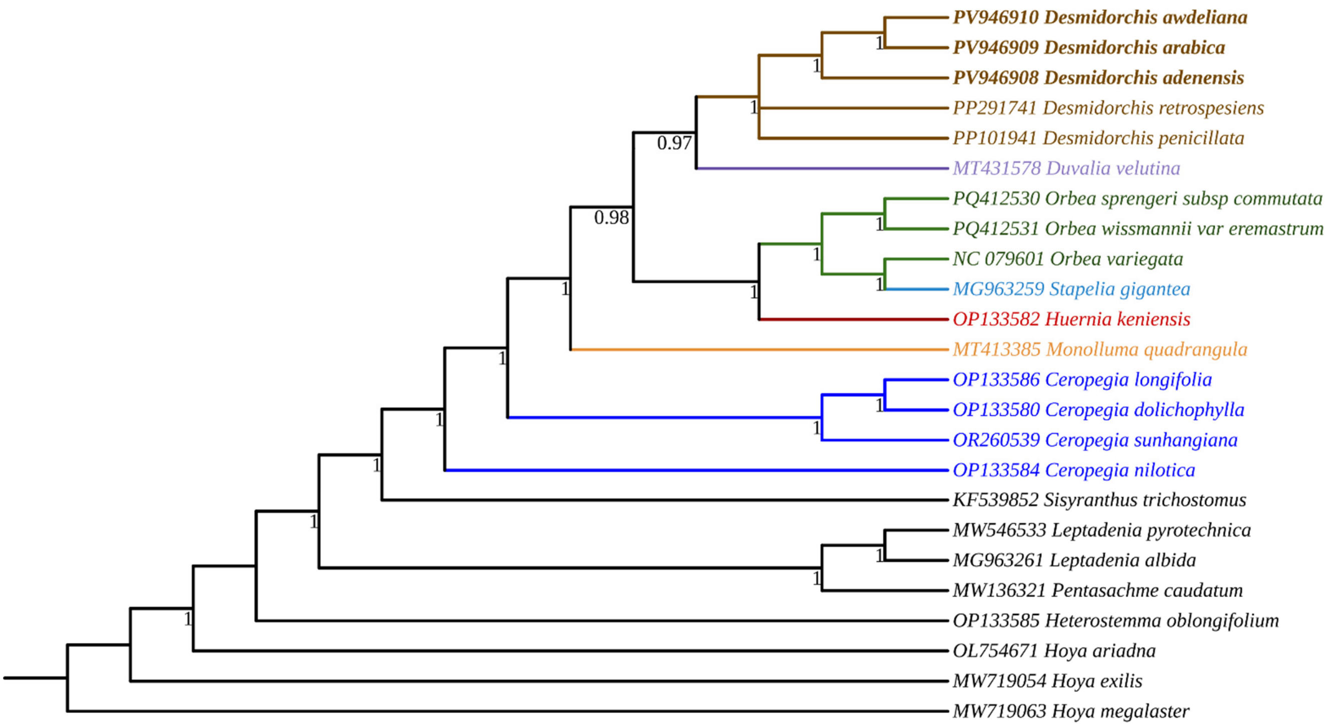

Figure S4. Bayesian inference (BI) tree of 24 Ceropegieae species and three outgroups based on 80 protein-coding genes. Bayesian posterior probabilities (PP) are shown above branches. Subtribe Stapeliinae genera are color-coded. The newly sequenced *Desmidorchis* species are highlighted in bold brown.

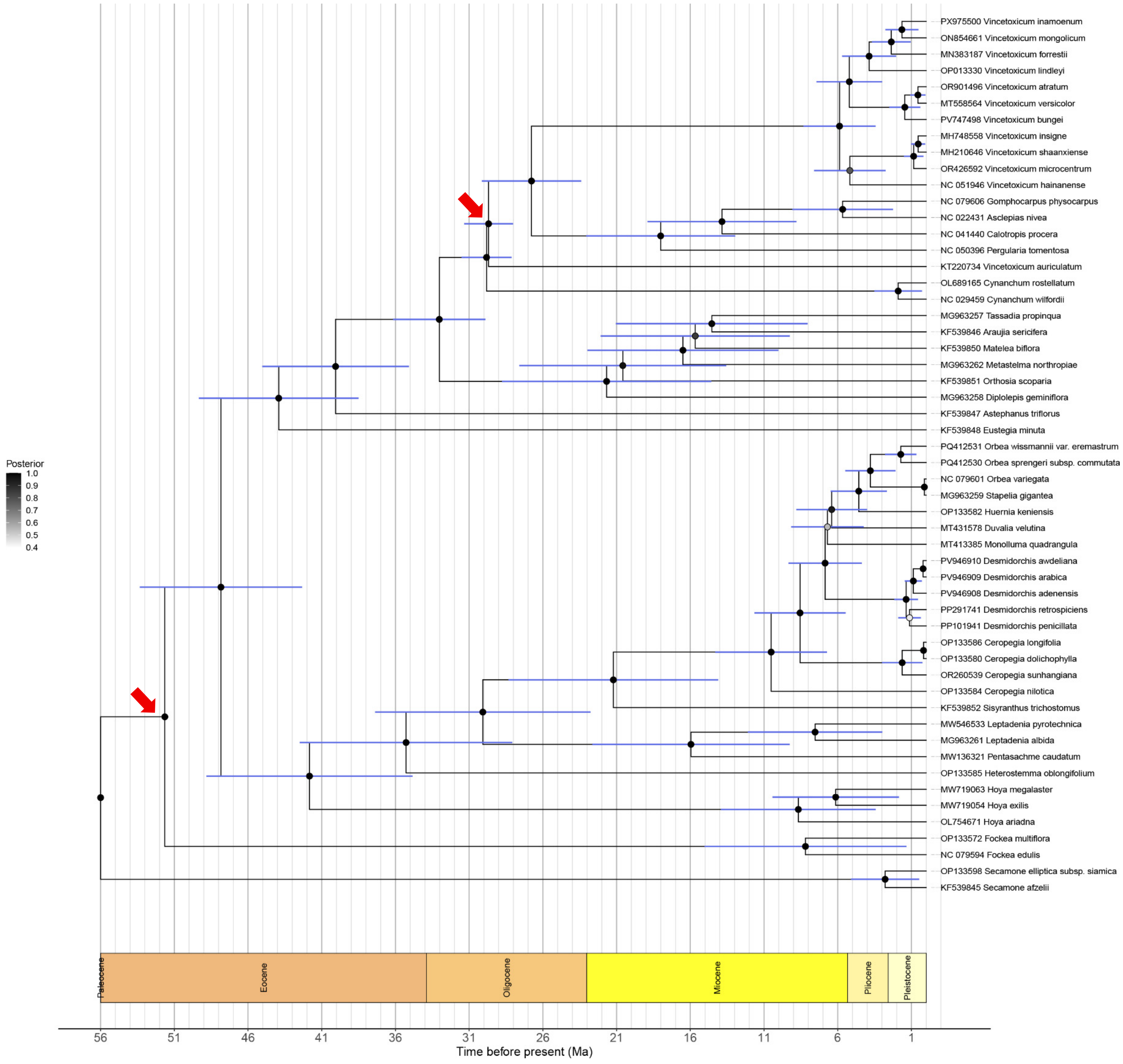

Figure S5. Maximum clade credibility chronogram of Asclepiadoideae based on 62 shared chloroplast protein-coding genes. blue bars indicate the 95% highest posterior density. Calibrated nodes are marked with red arrows
